# An efficient method for generation of bi-allelic null mutant mouse embryonic stem cells and its application for investigating epigenetic modifiers

**DOI:** 10.1093/nar/gkx811

**Published:** 2017-09-13

**Authors:** Cynthia L. Fisher, Hendrik Marks, Lily Ting-yin Cho, Robert Andrews, Sam Wormald, Thomas Carroll, Vivek Iyer, Peri Tate, Barry Rosen, Hendrik G. Stunnenberg, Amanda G. Fisher, William C. Skarnes

**Affiliations:** 1 Wellcome Trust Sanger Institute, Wellcome Genome Campus, Hinxton, Cambridge, CB10 1SA, UK; 2 MRC London Institute of Medical Sciences and Imperial College London, Hammersmith Hospital Campus, Du Cane Road, London, W12 0NN, UK; 3 Department of Molecular Biology, Faculty of Science, Radboud Institute for Molecular Life Sciences (RIMLS), Radboud University, 6525 GA, Nijmegen, The Netherlands; 4 Cardiff University School of Medicine, Heath Park, Cardiff, CF14 4XN, UK

## Abstract

Mouse embryonic stem (ES) cells are a popular model system to study biological processes, though uncovering recessive phenotypes requires inactivating both alleles. Building upon resources from the International Knockout Mouse Consortium (IKMC), we developed a targeting vector for second allele inactivation in conditional-ready IKMC ‘knockout-first’ ES cell lines. We applied our technology to several epigenetic regulators, recovering bi-allelic targeted clones with a high efficiency of 60% and used Flp recombinase to restore expression in two null cell lines to demonstrate how our system confirms causality through mutant phenotype reversion. We designed our strategy to select against re-targeting the ‘knockout-first’ allele and identify essential genes in ES cells, including the histone methyltransferase *Setdb1*. For confirmation, we exploited the flexibility of our system, enabling tamoxifen inducible conditional gene ablation while controlling for genetic background and tamoxifen effects. *Setdb1* ablated ES cells exhibit severe growth inhibition, which is not rescued by exogenous Nanog expression or culturing in naive pluripotency ‘2i’ media, suggesting that the self-renewal defect is mediated through pluripotency network independent pathways. Our strategy to generate null mutant mouse ES cells is applicable to thousands of genes and repurposes existing IKMC Intermediate Vectors.

## INTRODUCTION

Pluripotent stem cells have attracted much attention due to their relevance for regenerative medicine (1). Mouse embryonic stem (ES) cells are pluripotent cells derived from the inner cell mass of blastocyst stage embryos that typically retain their normal diploid karyotype, are able to contribute to all embryonic lineages including germ cells and provide a faithful *in vitro* model of pre-implantation embryonic cells (2). Mouse ES cells are highly amenable to genetic manipulation (3), can be grown in sufficient numbers for conducting genome-wide assays and can be directed to differentiate into a wide variety of more mature cell types. Many aspects of gene function can be readily studied in ES cells or their cultured derivatives, without the need for costly and time-consuming generation and maintenance of mutant mouse models. Thus, ES cells provide an excellent model system for the elucidation of pathways required for cellular, developmental and disease processes.

A number of approaches have been used to achieve gene depletion or ablation in mouse ES cells. These include chemical (e.g. ENU) and transposon-mediated mutagenesis (4,5), RNA inactivation (RNAi) (6), gene trapping (7,8), gene targeting (4,9), targeted trapping (10,11), Zinc-Finger Nucleases (ZFN) and transcription activator-like effector nucleases (TALENs) (12) and CRISPR-Cas9 endonuclease systems (13,14). In functional genetic studies, residual gene activity often occurs when using RNAi gene knockdown techniques, which can mask a discernable phenotype. Accordingly, it is advantageous to inactivate both alleles of the gene of interest in ES cells to facilitate detection of a phenotype. One approach is to produce a library of random insertional mutations in Bloom-deficient ES cells (15) and select for populations of homozygous mutant cells following mitotic recombination (16,17). Insertional mutagenesis has also been applied in haploid mouse ES cells (18,19), obviating the need to select for bi-allelic null mutational events. Such libraries are ideal for forward genetic screens where there is a strong selectable phenotype (e.g. resistance to a drug or toxin, gain of ES self-renewal in differentiation-permissive culture); however, genome coverage is limited by the random nature of the insertional mutagenesis strategy. Recently, the first individually cloned CRISPR-Cas9 genome-wide arrayed sgRNA library for the mouse was described (20) which should facilitate candidate gene validation upon its application to forward genetic screens in mouse ES cells.

Bi-allelic mutations for complete gene inactivation at a desired locus (i.e. reverse genetics) can be generated in a variety of ways in mouse ES cells. In recent years, genome-editing techniques have emerged which utilize site-specific or RNA-guided nucleases capable of inducing null mutations in specific genes and which can generate bi-allelic constitutive null ES cells. In applications of ZFN and TALENs, protein engineering of the site-specific nucleases is required, validation of which can be time consuming (12). In applying the CRISPR-Cas9 endonuclease system, the intial step to design and synthesize a guide RNA is more tractable (12–14,21). However there is concern about off-target effects and the methodology for analyzing and reporting CRISPR-Cas9 off-target activity remains to be standardized (3,22–24). Schick *et al.* (25) reported that the incidence of random genomic insertions of CRISPR-Cas9-based vectors was 13-fold higher than that obtained when using conventional gene targeting approaches, which are typically low, on the order of 2%. While CRISPR-Cas9 nuclease technology continues to develop and improve, arguably a reliable and extensively validated method to generate null mutations in mouse ES cells remains gene targeting using homologous recombination. Some targeted loci are amenable to direct selection of loss of heterozygosity events in alleles containing the neomycin selection cassette by treatment with high concentrations of G418, thereby generating homozygous mutant ES cells (26); however success using this approach is unpredictable. Targeting both alleles of a gene to generate bi-allelic null ES cells can be achieved by recycling the vector used for initial targeting of the first allele (27,28) or by using two vectors with different selectable markers (29). Recycling the vector is often inefficient due to preferential retargeting of the first allele because of its’ greater homology to the vector. We describe a novel strategy to generate bi-allelic null ES cells utilizing the two vector approach, by taking advantage of the extensive vector reagent and mutant mouse ES cell line resources of the International Knockout Mouse Consortium (IKMC) and International Mouse Phenotyping Consortium (IMPC) (30–33) and designing a new vector for highly efficient ‘targeted trapping’ of the second allele.

The IKMC undertook projects to systematically knockout all protein-coding genes in the mouse genome using methods of high-throughput recombineering and gene targeting by homologous recombination (34–36). That work is complete, with targeted heterozygous knockouts available through IMPC public resources for >18 500 genes in C57BL/6 ES cells (25,31,37) and ∼5000 strains of mutant mice generated which have uncovered novel phenotypes of known and previously uncharacterized genes (37–41). This platform represents a valuable cost-effective resource for the biomedical research community. A major fraction of the IKMC resource, comprising the EUCOMM and KOMP-CSD projects (31,35), was based on the use of a promoterless or promoter-driven targeting vector to generate the ‘knockout-first, conditional-ready’ *tm1a* allele (42). These alleles were designed to initially disrupt gene function by splicing to a *lacZ* gene trapping element, and can be modified through the action of Flp or Cre recombinases to generate conditional and deletion alleles (28). En route to constructing the final targeting vector, Gateway-adapted Intermediate Vectors were produced by recombineering and custom Gateway-adapted targeting elements (positive and negative selection cassettes) were incorporated into the Intermediate Vector in an *in vitro* Gateway exchange reaction to produce the final targeting vector (35). These Intermediate Vectors were purposely built in a modular way to enable the construction of other useful alleles in ES cells, to extend the utility of the resource (37,43).

In this study, we took advantage of the flexibility of the EUCOMM and KOMP-CSD vector systems to design new conversion cassettes, which we used together with the extensive library of EUCOMM and KOMP-CSD Intermediate Vectors, to rapidly assemble new insertion vectors for ‘targeted trapping’ (10) of the second allele, using the Gateway cloning system. We developed a highly efficient method for generation of null mutant mouse ES cells, using existing IKMC resource ‘knockout-first conditional-ready’ heterozygous mutant ES cells and constitutively inactivating the second allele with our new insertion-type targeted trapping vectors built using the modular IKMC resource Intermediate Vectors. To demonstrate the utility of our gene ablation approach in mouse ES cells, we applied the method to 14 genes with chromatin binding and/or epigenetic regulatory function, performing second allele targeting in cell lines that already contain one corresponding ‘knockout-first’ null allele made through promoterless vector targeting in the IKMC pipeline. A useful feature of our design strategy is the ability to reinstate gene expression in our bi-allelically targeted ES cells by reverting one mutant allele with Flp recombinase, a powerful way to demonstrate phenotype specificity via restored molecular function. We illustrate these features in detail using two genes encoding epigenetic factors as examples: *Cbx1* and *Jarid2*.

Notably, we designed our bi-allelic targeting strategy such that integration events of the second vector re-targeting the first allele are selected against early in the cell culture process, and hence the inability to obtain clones during screening likely indicates the gene is essential in ES cells. Interestingly, we failed to recover bi-allelic targeted clones for three genes in our targeting experiments, including the histone methyltransferase encoding *Setdb1* (also known as *Eset/Kmt1e*). As an additional demonstration of the flexibility and application of our method, we used an inducible conditional strategy (28) with the same IKMC vectors and cell lines but employing them in a different vector targeting order, combined with our novel insertion-type targeted trapping vector, to inducibly ablate *Setdb1* in ES cells. These experiments demonstrate that *Setdb1* is indeed an essential gene in undifferentiated mouse ES cells. We explore the functional consequences of inducible *Setdb1* depletion, observing severely reduced growth and increased differentiation of ES cells, leading us to investigate whether forced reinstatement of naïve pluripotency could rescue these effects. In summary, we describe several applications of our bi-allelic gene targeting strategies for complete gene ablation in mouse ES cells using our novel insertion-type targeted trapping vectors, built upon the modular and flexible IKMC public resource. Taken together, these flexible tools offer biomedical researchers an opportunity to investigate gene function in mouse ES cells using a gene knockout approach that is highly efficient and requires no vector design work.

## MATERIALS AND METHODS

### Preparation of vector components

Details on EUCOMM and KOMP-CSD Intermediate Vector designs (35) obtained from public IKMC resources, primers, and reading frames for pI_hygGFP insertion vector construction used in this study are listed in Supplementary Tables S1, S3 and S4. Additional information on IKMC reagents can be obtained from the IMPC web portal (33) (https://www.mousephenotype.org). DH10B *Escherichia coli* containing individual Intermediate Vectors were obtained from the IKMC (35) and individual clones were expanded in liquid culture containing 10 μg/ml zeomycin and 50 μg/ml ampicillin. Intermediate Vector DNA was digested with AsiSI to assess quality and confirm the expected size of ∼15 kb. The pL1L2_RloxP plasmid was grown in DH5alpha *E. coli* with 20 μg/ml clonNAT. The pL3L4_hygGFP plasmids were grown in DB3.1 *E. coli* with 15 μg/ml kanamycin and 12.5 μg/ml chloramphenicol. All DNA for cloning was prepared using Qiagen plasmid midi kits.

### Generation of pI_hygGFP plasmids

Three-way Gateway (Invitrogen™) reactions were performed in individual microcentrifuge tubes using LR Clonase II Plus enzyme mix (Invitrogen) according to the manufacturer’s instructions. The following vector components were reacted in equimolar amounts overnight at 25°C: 200 ng Intermediate Vector, 40 ng pL1L2_RloxP plasmid and 100 ng pL3L4_hygGFP plasmid of the appropriate reading frame. After inactivation with Proteinase K, the reactions were brought to 20 μl volume with HPLC-grade water, were microdialyzed on 0.1 μm filter (VCWP Millipore) over HPLC-grade water for 1.5 h and the resulting liquid collected into a fresh tube and brought back up to 20 μl with HPLC-grade water. The dialyzed Gateway reaction was transformed into electrocompetant DH10B *E. coli* containing the pSC101_705Cre temperature inducible chloramphenicol resistant Cre-expressing plasmid (Gene Bridges), which had been induced as follows: the DH10B + pSC101_705Cre *E. coli* were cultured in LB broth with 17.5 μg/ml chloramphenicol for 24–30 h (to saturation) at the non-permissive temperature of 30°C. A total of 400 μl of the saturated culture was then used to seed a 20 ml short-term culture in LB broth with 17.5 μg/ml chloramphenicol at 30°C until the OD600 reached 0.4. Then the short-term culture was induced at 42°C for 30 min (to express Cre recombinase), with constant shaking, followed by cooling of the cells on ice. Cells were made electrocompetant by three fast washes in HPLC-grade water (cells were spun at 4000 rpm for 5 min at 4°C each time). All following steps were performed quickly and components were kept on ice. Following the final wash the *E. coli* cells were gently resuspended in 500 μl ice-chilled HPLC-grade water. For each transformation, 50 μl of the electrocompetant cells were gently mixed with 2 μl of the Gateway reaction, transferred to a pre-cooled electroporation cuvette (0.1 cm gap, BioRad GenePulser) and electroporated using EC1 setting (BioRad MicroPulser). For recovery culture, 500 μl of 37°C Super Optimal broth with Catabolite repression (SOC) media (ThermoFisher) was added per cuvette, mixed and transferred to a 14 ml conical tube and incubated with shaking for 1 h at 37°C. Up to 250 μl of the recovery culture was plated onto YEG agar plates containing 25 μg/ml kanamycin and 50 μg/ml streptomycin. Note that DH10B clones with the proper final targeting insertion vector (containing the gapped region) will only grow on YEG/kan/strep if the loxP-flanked ‘critical exon’ has been deleted by Cre activity, as loss of rpsL reverts DH10B to being streptomycin resistant. LR Clonase negative reactions can be used to assess background. Clones obtained from LR Clonase negative reactions that grow on YEG/kan/strep should contain the pL3L4_hygGFP backbone vector; the level of background will help determine the number of +LR Clonase colonies necessary to pick to obtain a clone with the proper final targeting insertion vector. We routinely picked 12 clones from each +LR Clonase reaction and performed Sanger sequencing analysis on Qiagen miniprepped DNA with primers LR (to confirm deletion of the critical exon region) and hygro_1R (to confirm the reading frame), on all clones followed by restriction enzyme digests (AsiSI cuts twice; PmeI should linearize) to confirm each vector.

Final insertion targeting vector (pI_hygGFP) DNA was prepared using a Qiagen plasmid midiprep or maxiprep kit and were linearized with PmeI before proceeding to ES cell electroporation. All other vector DNA used for ES cell electroporation was prepared using Qiagen plasmid maxi kits. pI_hygGFP vectors that contain additional PmeI site(s) within the genomic homology arms cannot be linearized intact and so should be eliminated from the potential vector construction pool. To classify vectors, we used bulk data underlying the iMITS component of the IMPC website (33) (https://www.mousephenotype.org/imits/targ_rep/alleles). We counted numbers of vector designs and corresponding gene loci represented within the promoterless and promoter-containing IKMC targeting vectors and analyzed their Intermediate Vector designs. Using genomic sequences of the 5′- and 3′-homology arms of the vector designs as extracted from GRCm38 genomic sequences, we classified vectors into those with or without extra PmeI sites(s) (GTTT/AAAC) (Supplementary Table S2a) and noted details of those genes represented by promoterless type IKMC vector designs (Supplementary Table S2b).

### Cell culture

All ES cells used in this study are murine. Wild type (WT) JM8 ES cells (44) and all JM8-based ES cell lines used or derived in this study, were cultured as described in IKMC protocols (35), without fibroblast feeders on tissue culture plates coated with 0.1% gelatin (Sigma) in phosphate-buffered saline (PBS), unless stated otherwise. ES cells were routinely cultured in ‘regular ES cell media’ (serum+LIF) containing Knockout DMEM (Gibco) supplemented with 10% fetal bovine serum (Invitrogen; pre-screened for supporting undifferentiated ES cell culture), 2 mM L-Glutamine (Gibco), 0.05 mM β-mercaptoethanol (Sigma or Gibco) and LIF (Chemicon/Millipore) according to manufacturer’s instructions. For experiments with cell lines once established and expanded, we added 1× penicillin–streptomycin (Gibco). For ES cell culture under ‘2-inhibitor’ (2i) conditions, we used a mixture of 75% 2i culture media (2,45) plus 25% ‘regular ES cell media’, all supplemented with LIF (Chemicon/Millipore) according to manufacturer’s instructions. Cells were plated onto tissue culture plastic previously coated in laminin (10 μg/ml in PBS; Sigma) for a minimum of 4 h in a 37°C tissue culture incubator followed by coating in 0.1% gelatin in PBS for at least 5 min at room temperature. The 2i culture media was a 1:1 mixture of Neurobasal and DMEM:F12 media (Gibco), to which was added 0.5× N-2 Supplement (Gibco), 0.5× B-27 Supplement (Gibco), 2 mM L-Glutamine, 0.1 mM β-mercaptoethanol, 1 μM PD032590 (Stemgent), 3 μM CHIR99021 (Stemgent) and 1× penicillin–streptomycin (Gibco). Cells were maintained in 75% 2i culture media plus 25% ‘regular ES cell media’ for several passages prior to use in experiments.

### Electroporation and ES cell line generation

To generate the doubly targeted *tm1a/tm2* mutant ES cells or the singly targeted *tm2/+* control lines, the *tm1a/^+^* or WT JM8 ES cells respectively, were electroporated with the relevant linearized pI_hygGFP vector using a 25-well cuvette electroporation system as described (35). Cell suspensions of ∼1 × 10^7^ cells in PBS and 2 μg of linearized vector were mixed immediately prior in a final volume of 120 μl, electroporated then allowed to rest for 20 min, then recovered into 20 ml of ES cell media without selective agents and plated onto 10 cm dishes. The following day selection using 120 μg/ml hygromycin B (Calbiochem) in media was started and continued until colonies were picked 10–14 days later, with daily media changes. Routinely, 24 colonies per electroporation were picked into 96-well plates and for subsequent culturing the concentration of hygromycin B was reduced to 100 μg/ml. Cells were split into replicate 96-well plates for archiving and preparation of genomic DNA for genotyping as described (35).

To revert the mutant *tm1a* allele to the pre-conditional *tm1c* allele, the recombinase flippase (Flp) was transiently transfected into ES cells by introducing a Flp encoding puromycin resistant plasmid (pCAGGs-FLPe) (46). A total of 50 μg of circular plasmid was combined with approximately 5 × 10^7^ ES cells in PBS in a final volume of 700 μl and electroporated in a 0.4 cm gap cuvette in a GenePulser electroporator (BioRad) at 250 V and 500 μF on high capacitance setting. Cells were allowed to rest for 20 min following electroporation and then the mixture was recovered into 20 ml of media without selective agents and equal volumes were plated onto two 10 cm dishes. Selection in 0.8 μg/ml puromycin (Sigma) was started 36–48 h after electroporation and was maintained for 3 days after which culture continued without any selective agents, with daily media changes throughout, until colonies became visible. A duplicate 10 cm colony plate was stained with X-gal in order to quantify the number of blue versus white colonies to determine an adequate number of colonies to pick for screening/genotyping, to increase efficiency. Blue stained cells indicate presence of the intact *tm1a* allele due to the expression of the β-galactosidase encoding lacZ transgene in the lacZ-neomycin phosphotransferase (β-geo) trapping cassette, as shown in Supplementary Figure S2e, whereas absence of blue stain in Flp recombinase treated cells likely indicates a desired reversion event whereby the betageo cassette located between two flippase recognition target (FRT) sites is excised by Flp recombinase, generating the *tm1c* allele. Colonies were picked into 96-well plates, expanded and replicated for further X-gal staining to identify unstained (white) clones, archiving and preparation of genomic DNA for genotyping. Expanded revertant clones of confirmed genotype were tested for puromycin sensitivity as subsequent genetic manipulations might involve the use of puromycin selection. We note however, that if a different targeted gene of interest is expressed at a low level, the initial X-gal stain visual screening method to detect a *tm1c* reverted allele may not be sensitive enough, in which case only the genomic DNA-based screening methods for genotyping the reversion event could be applied.

To generate the *Setdb1* inducible conditional ES cell lines, heterozygous ES cell lines with one WT and one *tm1a* allele were first transiently transfected with the pCAGGs-FLPe vector to generate *tm1c /+* revertant lines, as described above. These revertant lines were then transfected with the pI_hygGFP vector to generate *tm1c/tm2* doubly targeted ES cell lines. The final step utilized the Rosa26-CreERT2 knock-in plasmid to target a drug inducible mutant estrogen receptor Cre fusion protein into the ubiquitously expressed *Rosa26* locus (47,48). The *tm1c/tm2* (or *tm1c/^+^* as controls) ES cell lines were targeted with 2 μg of SfiI-linearized Rosa26-CreERT2 plasmid by electroporation in the 25-well cuvette as described above except that 5 × 10^6^ cells were used per electroporation. Cells were plated onto two 10 cm dishes at densities of 1 × 10^6^ or 5 × 10^5^ per plate and selection in 1 μg/ml puromycin was started 48 h after plating and continued until colonies were picked 10–12 days later, with daily media changes. Colonies were picked into 96-well plates for screening and polymerase chain reaction (PCR)-based genotyping. Expanded *Setdb1 tm1c/^+^* and *tm1c/tm2* ES clonal cell lines containing the Rosa26-CreERT2 cassette were treated for a defined period (48 h unless otherwise noted) with 0.8 μM hydroxytamoxifen (4′OHT, Sigma) to induce Cre activity and convert the *tm1c* allele to *tm1d* and then were subsequently cultured without 4′OHT for use in downstream assays. Concurrently, *tm1c/^+^* ES clonal cell lines with Rosa26-CreERT2 were cultured under identical conditions in 4′OHT, as controls due to possible effects of toxicity upon exposure to 4′OHT and/or Cre activity (49). We note that there is a variable but low level of recombination at the *Setdb1* locus occurring in the absence of Cre induction in some samples (e.g. refer to Supplementary Figure S7a and c). Such ‘leakiness’ is not uncommon with inducible systems and the CreERT2 cassette has compared favorably to other Cre induction cassettes in specific tests to quantify the extent and functional impact of background recombination *in vivo* in mice (47).

To generate ES cell lines constitutively expressing *Nanog*, we electroporated 30 μg of ScaI-linearized pPyCAG-Nanog-IRES-Blasticidin transgenic vector (gift from Jose Silva, University of Cambridge) into 5 × 10^7^ ES cells, using a GenePulser electroporator (BioRad) set at 800 V and 3 μF, in a final volume of 800 μl PBS in a 0.4 cm gap cuvette. Cells were plated on 10 cm gelatinized plates and selection in 6 μg/ml blasticidin (ThermoFisher) was started 48 h later and continued thereafter. Colonies were picked 9 days later into 96-well plates to establish stable cell lines and *Nanog* expression in a subset of four clones following expansion was confirmed by quantitative real time-polymerase chain reaction (qRT-PCR) analysis.

### Genomic DNA preparation and PCR genotyping

For screening ES cell clones, genomic DNA was prepared from 96 well plates as described (35). To confirm the genotype of expanded ES cell clones, genomic DNA was prepared from confluent cultures of 24 well plates using DNAzol (Invitrogen). Genotyping by long range PCR was done using either the Expand Long Template PCR System (Roche) with System 2 Buffer plus an extra 1.25 mM MgCl_2_ and the additives 1% DMSO and 67 mM Trehalose, or the SequalPrep Long PCR kit with dNTPs (Invitrogen), according to manufacturer’s instructions and the following cycling protocol was performed in 96 well format and heated lid: 94°C 3 min; 10 cycles of: 94°C 10–15 s, 70°C 30 s with −1°C per cycle, 68°C 6–7 min; 25 cycles of: 94°C 10–15 s, 60°C 30 s, 68°C 6 min plus add 20 s per cycle; 1 cycle: 7 min at 68°C (Roche Expand polymerase) or 72°C (SequalPrep polymerase); 4°C hold. Short range PCRs were done using Platinum Taq polymerase (Invitrogen) with the manufacturers recommended conditions. PCRs were done in BioRad Tetrad or Genomic Research Instrumentation Ltd (GRI) G-Storm thermocyclers.

### Genotyping strategies and PCR primers

The recombineering oligonucleotide primers from the IKMC gene targeting pipeline (35) used to generate the Intermediate Vectors which were used in this study are listed in Supplementary Table S4; the 5′ and 3′ ends of the genomic homology arms in each vector are denoted by the G5 and G3 primers respectively, whereas the U3 and D5 primers respectively denote the 5′ and 3′ boundaries of the loxP flanked region within the 3′ genomic homology arm. This information is included as it is critical for the genotyping strategy of targeted cells to identify genomic DNA sequence external to that which is contained in the targeting vectors, as one primer for PCR screening needs to be externally located.

**Figure 1.**
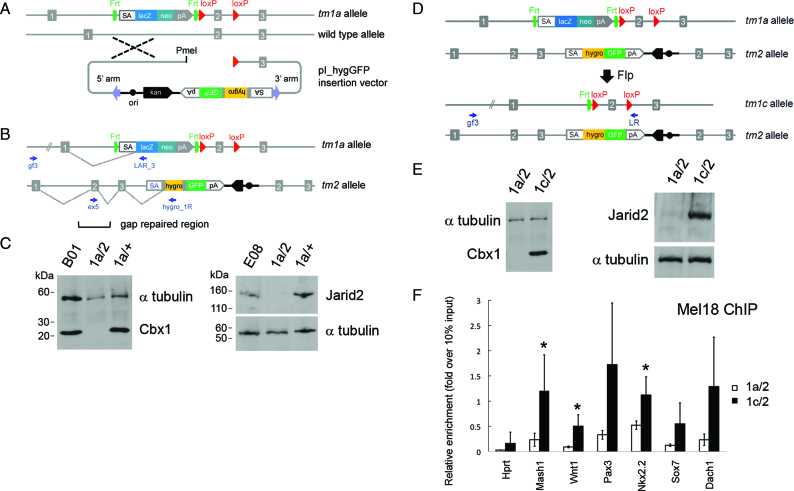
Schematic of allele structures in second allele targeted and revertant ES cells and cell line validation. (**A**) Insertion-type targeting vector pI_hygGFP for inactivation of the WT allele in ES cells heterozygous for a standard IKMC knockout-first allele (*tm1a*). (**B**) Structure of the bi-allelic locus after targeting the second allele. The *tm2* allele contains the pI_hygGFP targeting cassette and duplicated homology region, where exon 2 is re-generated by gap repair. (**C**) Western blots of Cbx1 and Jarid2 parental IKMC heterozygous ES cells (B01 and E08 lines), and examples of cell lines following pI_hygGFP electroporation including doubly targeted ES cells (*1a/2*) showing the absence of protein expression, and failed targeting events (*1a/+*). (**D**) Reversion from null mutant (*1a/2*) to conditional mutant (*1c/2*) by Flp recombinase. (**E**) Western blots of mutant (*1a/2*) and reverted (*1c/2*) Cbx1 and Jarid2 ES cell lines showing re-expression of protein. Primers for LR-PCR genotyping are indicated by small arrows and α-tubulin was used for Western blot loading controls. (**F**) Rescue of Polycomb PRC1 recruitment to PRC2 target genes in *Jarid2* revertant cell lines (*1c/2*), shown by reinstatement of Mel18 binding at known Jarid2-dependent gene promoter regions, assessed by chromatin immunoprecipitation (ChIP)-qRT-PCR. *Hprt* is a control locus known to be negative for PRC1 binding. Results show mean ± s.d. of three biological replicates (independent cell lines), where values are expressed as relative fold-enrichment over 10% input chromatin. Asterisks indicate statistically significant differences between *Jarid2* revertant (*1c/2*) and null (*1a/2*) cell lines (*P* < 0.05, one-tailed Student’s *t*-test).

Long-range PCR (LR-PCR) (35) was used to screen for targeted cells resulting from electroporations of the pI_hygGFP vectors into either heterozygous IKMC mutant cells (*tm1a/^+^*) or into WT cells as a control and genotypes of expanded clones were confirmed with the same primer sets, listed in Supplementary Table S3, prior to use in further experiments. We confirmed the presence of the ‘knockout first’ allele (*tm1a*) using a gene-specific forward primer external to the 5′ genomic homology arm (gf3) and a vector-specific reverse primer within the lacZ cassette (LAR_3). To detect targeting of the second allele (*tm2*) with pI_hygGFP vector, we used a gene-specific forward primer, ex5, that binds within the deleted genomic sequence region within the pI_hygGFP targeting vector (this region would undergo gap repair and therefore be present only in the targeted allele in the genome) and a vector-specific reverse primer in the hygromycin-GFP cassette, hygro_1R, as illustrated in Figure 1B and Supplementary Figure S2a. The size range of these LR-PCR products is 6–7 kb, as shown in Supplementary Figure S2b and c. Random insertions of the targeting vector are not detected with this primer combination.

ES cell clones obtained following transient Flp expression (which should convert the *tm1a* allele to the *tm1c* allele) were initially screened for loss of X-gal staining resulting from deletion of the FRT flanked lacZ-neomycin cassette and those clones negative for staining were genotyped by LR-PCR on genomic DNA using a locus specific external forward primer 5′ to the 5′ genomic homology arm of the vector, gf3 and a vector specific reverse primer, LR, which binds just 3′ to the 3′ loxP site of the *tm1a* and *tm1c* alleles, as shown in Figure 1D and Supplementary Figure S2a. These primers amplify the *tm1a* allele with an expected amplicon size of approximately 12 kb, whereas the amplicon size for the *tm1c* allele template with recombined FRT sites will be reduced to ∼7 kb, as shown in Supplementary Figures S2d and 5a left panel). No amplification occurs from the *tm2* allele since the LR reverse primer does not bind this template.

Revertant (*tm1c/^+^*) ES cell clones following targeting with the pI_hygGFP insertion vector (as we have demonstrated for *Setdb1*) were genotyped for targeting as described above, however, this assay will not distinguish between *cis* and *trans* targeting of the conditional (*tm1c*) allele. To detect undesirable *cis* targeting events, clones were further screened by LR-PCR using vector-specific primers, R1_amp_F2 and hygro_1R, which bind to the *tm1c* allelic and hygromycin gene sequences (contained in pI_hygGFP vector) respectively. This primer set will amplify a 6 kb fragment if the two integrated vectors are in *cis*; no product would result if they are in *trans* configuration, as shown in Supplementary Figure S5a middle panel.

For genotyping of Rosa26-CreERT2 knockin vector targeted ES cells, we inferred correct integration based upon successful functioning of the CreERT2 cassette upon 4′OHT induction, by the ability of Cre to recombine the loxP sites at the *Setdb1 tm1c* locus. This was done using short range PCR at the *tm1c* allele whereby clones with a functional integrated CreERT2 cassette would show a reduced amplicon size after exposure to Cre activity, turning the *tm1c* allele into *tm1d*, as shown in Supplementary Figure S5a right panel. Southern-blot and PCR-based genotyping strategies for Rosa26-CreERT2 vector targeting have been described, for use in further validation (47,48).

Each PCR described in this section was done in a separate reaction with one primer pair. All primer sequences are listed in Supplementary Table S3.

Descriptions of cell-based assays and microscopy, western blot analysis, gene expression analysis, ChIP, sequencing and ChIP-seq analyses and associated references are available in the Supplementary Materials and Methods.

## RESULTS

### Construction of insertion vectors for second allele targeting

Taking advantage of the modular design of IKMC targeting vectors (35), we re-configured Intermediate Vectors from the resource (Supplementary Table S1 and Figure S1a) into insertion-type vectors (4) for high efficiency ‘targeted trapping’ (10) of the second allele, applicable to expressed genes in mouse ES cells (Supplementary Figure S1). To this end we developed: (i) an adaptor module (pL1L2_RloxP; Supplementary Figure S1b) for the introduction of a rare PmeI restriction site for linearization and one loxP recombination site upstream of the ‘critical exon’ (35) and (ii) a series of hygromycin-GFP gene trap cassettes (pL3L4_hygGFP; Supplementary Figure S1c) in three reading frames, to replace the plasmid backbone. The reading frame is determined by the end phase of the most 3′ exon within the genomic homology region. Expression of the hygromycin resistance gene and GFP reporter is dependent on splicing to the endogenous gene to produce an in-frame fusion protein and ribosome-skipping elements (‘2A-like’ sequences) (35,50) were included in order to express multiple proteins from the same open reading frame and ensure optimal co-expression of the transgenes. Constructs are assembled in a three-part Gateway™ exchange reaction with the Intermediate Vector, the pL1L2 module and the pL3L4 trapping cassette (Supplementary Figure S1d) The final insertion-type targeted trapping vector pI_hygGFP is recovered by transformation into bacteria expressing Cre recombinase which deletes the floxed ‘critical exon’ (35) in the vector and creates a gap in the genomic homology (Supplementary Figure S1d). This gap facilitates the use of PCR-based genotyping to specifically screen for the targeted mutant allele, since a primer annealing site within the deleted region will be regenerated only upon gap repair at the locus in ES cells following homologous recombination (51) and clones in which random integration of the vector occurred will be PCR-negative.

### Generation and validation of epigenetic regulator bi-allelic mutant ES cell lines

Insertion-type targeted trapping pI_hygGFP vectors were electroporated into the corresponding heterozygous IKMC ‘knockout first’ *tm1a/^+^* ES cells with the aim of generating bi-allelic null mutants (Figure 1A). The insertion targeting vectors pI_hygGFP are prepared by linearization with PmeI restriction enzyme and electroporated into IKMC targeted heterozygous ES cells that contain the ‘knockout-first’ allele (*tm1a*) (35) (Figure 1A). The presence of an additional PmeI restriction site within the IKMC Intermediate Vector genomic homology arms would preclude its’ use as a pI_hygGFP targeting vector in our system and this should be checked prior to final vector construction. Our analysis of IKMC Intermediate Vectors showed that 11.5% of vectors based on the IKMC ‘knockout-first’ promoterless cassette design, are not suitable for this reason (Supplementary Table S2a). Our bi-allelic ‘targeted trapping’ method was designed for application to genes expressed in mouse ES cells. Our strategy is therefore applicable to all genes represented with a KOMP-CSD or EUCOMM ‘knockout-first’ *tm1a* allele from a promoterless targeting vector, since that class of vectors specifically targets expressed genes (35). This set of 2257 promoterless vector designs for *tm1a* allele generation represents 1377 and 837 genes from the KOMP-CSD and EUCOMM pipelines respectively, noting that some genes have more than one vector design associated with them (Supplementary Table S2b) (31,33).

**Table 1.** Targeting frequencies following electroporation of insertion vectors pI_hygGFP into WT JM8 (parental or subcloned N4 or F6 lines as indicated) and heterozygous mutant *tm1a/^+^* (knockout first) ES cells, selection in hygromycin and screening/genotyping by long range-PCR on genomic DNA

Targeted gene (MGI symbol)	ES cell line/allele used for targeting	ES cell clone ID	Number of colonies screened	% clones targeted	MGI name for pI_hygGFP targeted allele (tm2)
***Brd8***	JM8.N4		24	96	*Brd8^tm1Wcs^*
	JM8.N4/*Brd8 ^tm1a(EUCOMM)Wtsi/+^*	E10	22	100	
***Cbx1***	JM8.N4/*Cbx1^tm1a(EUCOMM)Wtsi/+^*	B01	24	45.8	*Cbx1^tm1Wcs^*
***Ddx27***	JM8.N4		24	100	*Ddx27^tm1Wcs^*
	JM8.F6/*Ddx27 ^tm1a(KOMP)Wtsi/+^*	E01	6*	0	
***Dot1l***	JM8 parental/*Dot1l ^tm1a(KOMP)Wtsi/+^*	D02	24	54.2	*Dot1l^tm1Wcs^*
***Epc2***	JM8 parental/*Epc2 ^tm1a(EUCOMM)Wtsi/+^*	B12	24	45.8	*Epc2^tm1Wcs^*
***Ing3***	JM8.N4		24	79	*Ing3^tm1Wcs^*
	JM8.N4/*Ing3 ^tm1a(EUCOMM)Wtsi/+^*	E07	16	81	
***Jarid2***	JM8 parental/*Jarid2 ^tm1a(KOMP)Wtsi/+^*	A08	24	62.5	*Jarid2^tm1Wcs^*
	JM8 parental/*Jarid2 ^tm1a(KOMP)Wtsi/+^*	E08	24	62.5	
***Kdm4c***	JM8.N4		12	100	*Kdm4c^tm1Wcs^*
	JM8.F6/*Kdm4c ^tm1a(KOMP)Wtsi/+^*	D03	24	75	
***Mier1***	JM8.N4		24	66.7	*Mier1^tm1Wcs^*
	JM8.N4/*Mier1 ^tm1a(EUCOMM)Wtsi/+^*	A04	24	66.7	
***Phf20***	JM8.N4		24	62.5	*Phf20^tm1Wcs^*
	JM8.N4/*Phf20 ^tm1a(EUCOMM)Wtsi/+^*	F07	0	0	
***Setdb1***	JM8.N4		16	100	*Setdb1^tm1Wcs^*
	JM8.N4/*Setdb1 ^tm1a(EUCOMM)Wtsi/+^*	D08	40	0	
***Smyd5***	JM8.N4		16	62.5	*Smyd5^tm1Wcs^*
	JM8.N4/*Smyd5 ^tm1a(EUCOMM)Wtsi/+^*	G01	24	25	
***Supv3l1***	JM8.N4		24	50	*Supv3l1^tm1Wcs^*
	JM8.N4/*Supv3l1 ^tm1a(EUCOMM)Wtsi/+^*	B08	24	70.8	
***Kmt5b***	JM8 parental/*Kmt5b ^tm1a(KOMP)Wtsi/+^*	C01	24	16.7	*Kmt5b^tm1Wcs^*

*Only a low number of colonies were obtained; all were screened.

Following homologous recombination with the WT allele, the entire pI_hygGFP vector is integrated into the genome in a single crossover event, placing the hygromycin-GFP cassette downstream of the genomic homology region in the vector and duplicating genomic sequences contained in the vector, thereby generating the second mutant allele (*tm2*) (Figure 1B). Conversely, homologous recombination *in cis* into the ‘knockout-first’ allele (*tm1a*) is not expected to give rise to hygromycin-resistant clones, as downstream gene expression in the locus is blocked by the upstream promoterless lacZ-neomycin trapping cassette. Hence, only bi-allelic targeted events are recovered from experiments introducing the final PmeI-linearized pI_hygGFP vector into IKMC heterozygous mutant cell lines containing the *tm1a* allele at the same locus, making the entire process highly efficient. Moreover, absence of bi-allelic targeted clones indicates the gene is likely essential for normal ES cell growth.

LR-PCR (35) was used to screen typically 24 ES cell clones per gene (Table 1; Supplementary Figure S2a–c; Supplementary Tables S3 and S4). We recovered doubly targeted ES cell clones (*tm1a/tm2*) for 11 of the 14 epigenetic regulatory genes we attempted to mutate, with an average targeting efficiency of 60%, ranging from 17–100% (Table 1). For clarity, we refer to the pI_hygGFP targeted mutant allele as ‘*tm2*’ to denote the second targeted mutation (Figure 1B), however the official Mouse Genome Informatics (MGI) allele names for each gene mutation follow standard nomenclature guidelines and are listed in Table 1. In parallel, WT ES cells were electroporated with pI_hygGFP vectors as a positive control for 9 of our 14 loci, and for this set we achieved an average targeting efficiency of 80%, with a range from 50–100% (Table 1). Doubly targeted ES lines were not recovered for three genes: *Ddx27, Phf20* and *Setdb1*, despite the fact that we could readily target these genes in WT ES cells with the corresponding pI_hygGFP vector to obtain heterozygous cell lines, validating our vectors and indicating that these genes are essential for ES cells. We observed a lower bi-allelic targeting efficiency in *Smyd5* heterozygous mutant cells (25%) than in WT cells (62.5%), suggesting decreased cell viability of null ES cells. We also observed a low targeting efficiency for the *Suv420h1* (also known as *Kmt5b*) gene (17%), however in this case we did not target WT ES cells for comparison.

We chose two doubly targeted viable cell lines, *Jarid2* and *Cbx1*, for further validation and as an example of the flexibility of our approach (Figure 1 and Supplementary Figure S2). Western blots confirmed the absence of full length protein in each line (Figure 1C and Supplementary Figure S2d). As we employed a targeted trapping (10) strategy, fusion transcripts will be generated from each mutant allele which are predicted to lead to the expression of truncation products, which are often unstable. The *Cbx1 tm2* allele is predicted to form a 172 aa (20 kDa) truncated protein resulting from splicing of exons 1–4 to the splice acceptor site of the vector, while the *Jarid2 tm2* allele is expected to give rise to a truncated protein of 97 aa (11 kDa) encoded by exons 1 and 2. Truncated products for *Cbx1* and *Jarid2* doubly targeted loci were not detected in western blotting with antibodies that would recognize the predicted truncation products, indicating that these truncated products are unstable and the mutant ES cells are indeed null. We did not observe any overt phenotypes of *Cbx1* or *Jarid2* null mutant ES cells during routine culturing.

### Reversion of Jarid2 and Cbx1 mutant ES cells

To confirm causality in a loss-of-function model, it is necessary to re-introduce WT protein and rescue the phenotype. We genetically reverted the null ‘knockout-first’ allele (*tm1a*) to a conditional allele (*tm1c*) (35) which is designed to express full-length protein, by excising the lacZ-neomycin trapping cassette with Flp recombinase through transient transfection of the pCAGGs-FLPe vector (46), leaving a single FRT site and two loxP sites flanking the ‘critical exon’ (Figure 1D and Supplementary Figure S2a). ES cells containing the *tm1a* allele stain blue when exposed to X-gal due to the presence of the lacZ gene in the trapping cassette (Supplementary Figure S2e). Following a ‘blue/white’ pre-screen for X-gal staining to isolate negative ES cell clones that have lost the lacZ gene, clones were genotyped by LR-PCR on genomic DNA to detect the product size reduction resulting from excision of the lacZ-neomycin cassette, indicating successful allelic reversion of *tm1a* to *tm1c* (Supplementary Figure S2f). Allelic reversion at the *Jarid2* and *Cbx1* loci was confirmed by Western blotting on genotyped clonal cell lines showing restored protein expression (Figure 1E and Supplementary Figure S2d). To elucidate possible downstream target genes and pathways affected by gene ablation, we compared gene expression profiles in *Cbx1* and *Jarid2* revertant cell lines (*tm1c/tm2*) with corresponding null mutant ES cell lines (*tm1a/tm2*). For *Cbx1*, only 20 genes showed altered expression (*P* < 0.05), with 8 genes upregulated and 14 downregulated in revertant compared to null cells (Supplementary Table S5). In contrast, our comparison of gene expression in *Jarid2* null and revertant cells yielded 242 upregulated and 212 downregulated genes in revertant compared to null ES cells (*P* < 0.05) (Supplementary Table S6). Gene Ontology (GO) analysis of the genes upregulated upon *Jarid2* reversion identified an enrichment of categories relating to RNA polymerase II transcription factor activity, the nucleosome and cell differentiation and developmental pathways (*P* < 0.05; Supplementary Figure S3a). Analysis of published target gene datasets for factors involved in ES cell regulation (Supplementary Materials and Methods) was performed on the *Jarid2* revertant versus null expression dataset and revealed significant differences in expression of targets of the Polycomb PRC2 component Suz12 and histone H3K27-trimethyl marked genes and targets of the pluripotency transcription factors Nanog, Sox2, Nac1, Dax1 and Cnot (*P* < 0.01; Supplementary Figure S3b). Functional reversion of a known Jarid2 phenotype at the molecular level was demonstrated by showing restored binding of Mel18 (Polycomb PRC1 component) at known Polycomb PRC2 target gene promoters (52) in *Jarid2* revertant ES cells, whereas absence of binding is observed as expected in *Jarid2* null ES cells, using ChIP coupled with qPCR (Figure 1F and Supplementary Figure S4).

### Generation and functional analysis of inducible conditional *Setdb1* mutant ES cell lines

**Figure 2.**
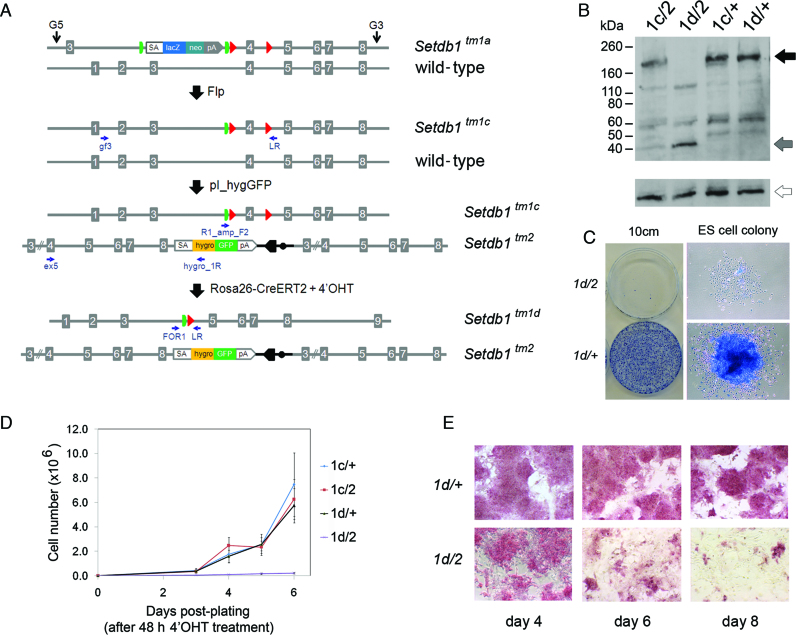
Generation and functional analysis of inducible conditional *Setdb1* mutant ES cell lines. (**A**) Allelic structures and workflow of vector electroporations. IKMC heterozygous ‘knockout-first’ (*tm1a/^+^*) ES cells are treated with Flp recombinase to generate a conditional allele (*tm1c*) and targeted with pI_hygGFP to generate conditional/null (*tm1c/tm2*) cells. Cre-ERT2 is then introduced by targeting vector knockin into the *Rosa26* locus. Upon treatment with 4′OHT, Cre recombinase activity removes the floxed ‘criticial exon’ of the *tm1c* allele to generate bi-allelic null cells (*tm1d/tm2*). Primers for PCR genotyping are noted as small arrows. (**B**) Western blots showing absence of full-length Setdb1 protein (black arrow) in the *Setdb1 tm1d/tm2* mutant ES cell lines after treatment with 4′OHT. A 39 kDa truncation product is generated from the *tm2* allele (gray arrow). α-tubulin was used as a loading control (open arrow). (**C**) *Setdb1 tm1d/tm2* mutant ES cells plated at low density (1 × 10∧3 cells per 10cm dish; following a 48 h 4′OHT treatment period) are unable to form colonies of undifferentiated ES cells, whereas control *Setdb1 +/tm1d* heterozygous ES cells (4′OHT treated and plated concurrently) exhibit normal undifferentiated ES cell colony morphology; a representative example is shown. Cells were stained with methylene blue 8 days after plating. (**D**) Growth of 4′OHT treated *Setdb1 tm1d/tm2* null ES cells is severely compromised, while 4′OHT treated control *Setdb1 +/tm1d* heterozygous ES cells retain robust growth characteristics similar to non-treated *Setdb1 tm1c/^+^* and *tm1c/tm2* cells. Cells were plated following a 48 h 4′OHT treatment and counted at the time intervals indicated. Data points are the mean of three biological replicates (independent cell lines), error bars indicate s.d. (**E**) 4′OHT-treated *Setdb1 tm1c/tm2* ES cells start to differentiate by 6 days after treatment and gradually lose alkaline phosphatase (AP) activity, while treated control *Setdb1 +/tm1d* ES cells retain AP activity and normal ES cell morphology.

We did not recover bi-allelic targeted ES cell clones for *Ddx27, Phf20* and *Setdb1* (Table 1), even though our pI_hygGFP vectors for these genes efficiently target WT ES cells. This suggested that these genes are required for ES cell survival since our bi-allelic targeting system was designed to select against re-targeting the ‘knockout-first’ (*tm1a*) allele. To explore this further, we made use of the flexibility of our system and employed an inducible conditional gene ablation strategy, involving three vector transfection steps followed by drug induction, to verify that the histone H3K9 methyltransferase *Setdb1* is an essential gene in ES cells (Figure 2 and Supplementary Figure S5). First, the ‘knockout-first’ allele (*tm1a*) (35) of *Setdb1* heterozygous IKMC ES cells was converted to a conditional allele (*tm1c*) with Flp recombinase (pCAGGs-FLPe vector). Second, the WT allele was targeted with the pI_hygGFP vector to generate *Setdb1 tm1c/tm2* ES cells. Third, inducible Cre recombinase (Cre-ERT2 knock-in vector) was introduced into the ubiquitously expressed *Rosa26* locus by gene targeting. Upon induction with 4′-hydroxytamoxifen (4′OHT), Cre is activated, deleting the ‘critical exon’ in the *tm1c* allele to generate null (*tm1d/tm2*) ES cells (Figure 2A). Following the first and second allele modification steps, clones were genotyped by long-range genomic PCR, whereas after the final step of Cre vector knock-in and induction, the clones were genotyped by short-amplicon genomic PCR (Supplementary Figure S5a). Inducible conditional *Setdb1 tm1c/^+^* heterozygous cells were generated concurrently as a control by collecting clones following the first step of Flp recombinase treatment and then proceeding directly to knock-in the Cre vector (Figure 2A and Supplementary Figure S5a). In order to control for potential changes due to drug treatment alone (49), for most experiments we compared ‘experimental’ *tm1c/tm2* (becoming *tm1d/tm2*) with ‘control’ *tm1c/^+^* (becoming *tm1d/^+^*) ES cells at timepoints before and after concurrent 4′OHT treatment.

Following 48 h treatment of bi-allelic inducible conditional mutant ES cell clones (*tm1c/tm2*) with 4′OHT, full-length Setdb1 protein is absent in *Setdb1* mutant (now *tm1d/tm2*) nuclear extracts (Figure 2B). The *Setdb1 tm2* allele removes exon 4 to induce a frameshift that is predicted to lead to 353 aa product, consistent with an observed 39 kDa truncation product in extracts containing this mutant allele (Figure 2B). The truncation occurs within the N-terminal Tudor domain (53) and the mutant product is missing the methyl-CpG DNA-binding motif, pre-SET and SET domains (54); therefore, the truncation product is expected to be non-functional. GFP expression from the *tm2* allele is detectable in these cells, as expected (Supplementary Figure S5b and c). To test whether *Setdb1* is an essential gene in ES cells, we performed stringent growth assays. *Setdb1* null mutant cells are unable to form normal ES cell colonies under clonal growth conditions (Figure 2C) and exhibit a severely impaired growth rate (Figure 2D). In bulk culture, *Setdb1* null ES cells appear normal up to 4 days after starting 4′OHT treatment, exhibiting strong alkaline phosphatase (AP) activity (Figure 2E), high levels of the pluripotency transcription factor Oct4 (also known as Pou5f1) protein expression and global histone H3K9 trimethylation (me3) (Supplementary Figure S5d). However by day 6, *Setdb1 tm1d/tm2* ES cells begin to exhibit a flattened morphology and are mostly differentiated by day 8, with very few AP positive cells remaining (Figure 2E). As with any inducible system, there is a possibility that a small number of non-recombined ‘escaper’ cells (genotype *tm1c/tm2*) remain following 4′OHT treatment when most cells are expected to have been converted to genotype *tm1d/tm2*. Based on our *Setdb1* mutant ES cell colony assay data (Figure 2C), we estimate having <1 in 1000 ‘escaper’ cells, since we rarely observed a normal ES colony (dense core of tightly packed cells strongly staining with methylene blue which would likely represent the non-recombined *tm1c/tm2* genotype) appearing after plating 1000 single 4′OHT treated cells in our replicates. To ensure that all cells in the culture have a *tm1d/tm2* genotype, for non-essential genes, we recommend that researchers subclone their cell lines following 4′OHT treatment prior to performing downstream assays.

### Gene expression and ChIP-seq analysis of inducible conditional *Setdb1* mutant ES cell lines

**Figure 3.**
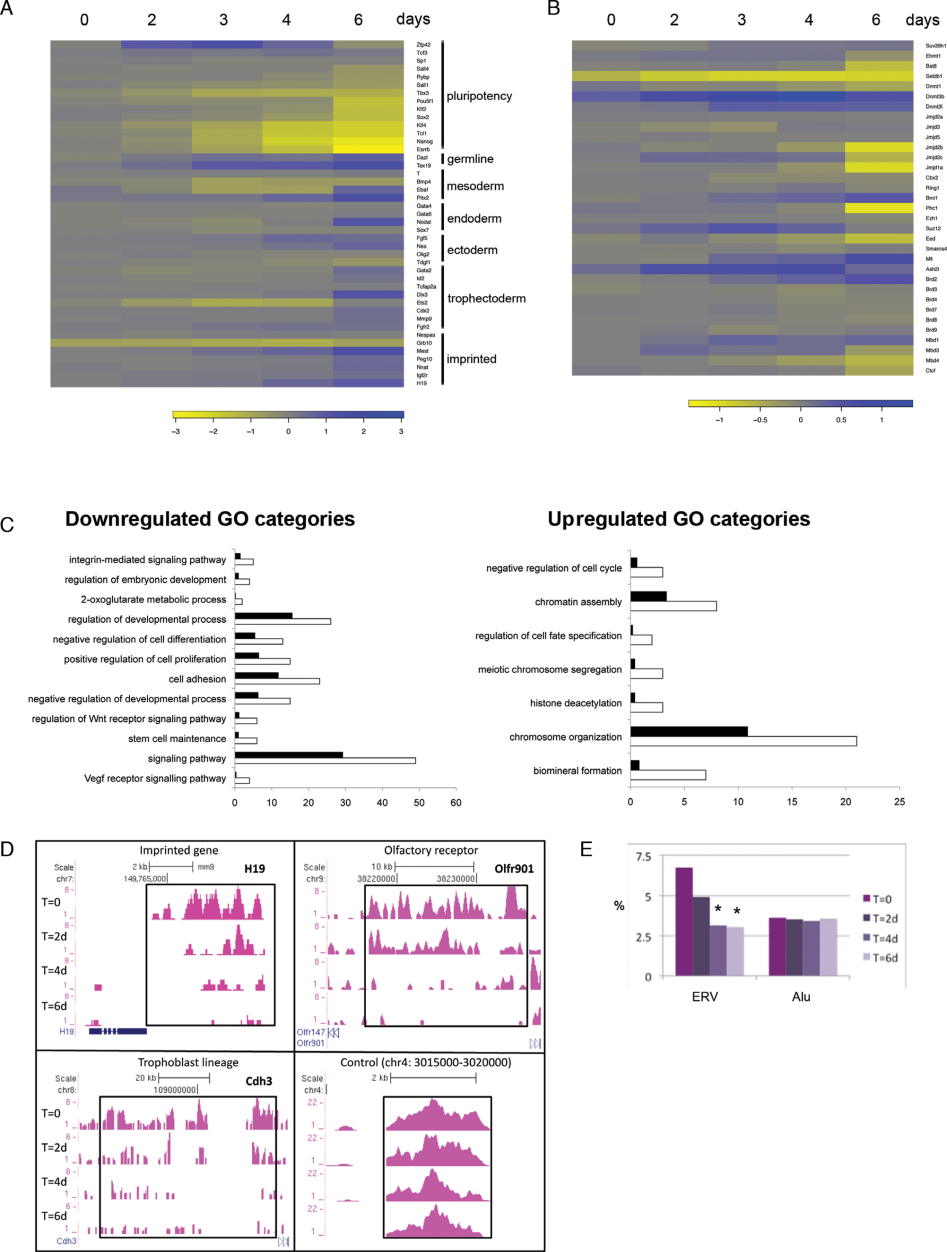
Timecourse analyses of gene expression and H3K9me3 histone modification marks in inducible conditional *Setdb1 tm1c/tm2* mutant ES cells compared to control *Setdb1 tm1c/^+^* ES cells following 4′OHT treatment and conversion of the conditional *tm1c* allele to the null *tm1d* allele. (**A**) Heatmap of changes in gene expression of pluripotency associated genes, lineage markers and imprinted genes by DNA microarray, following Setdb1 ablation. (**B**) Heatmap shows complex changes in expression of chromatin and epigenetic factors, in *Setdb1* null ES cells. Key to log-fold-change values are below each heatmap. Negative values, indicating downregulation in Setdb1 null ES cells, are shown in yellow; positive values, indicating upregulation in Setdb1 null ES cells, are shown in blue. (**C**) GO Biological Processes analysis of up- or downregulated genes at a single timepoint early after Setdb1 ablation in ES cells. Categories enriched in *Setdb1 tm1d/tm2* mutant versus *+/tm1d* control ES cell gene expression datasets after a 2-day 4′OHT treatment (same datatset as day 2 in heatmaps) are shown. White bars represent the number of genes observed while black bars represent the number of genes expected by chance in each GO category (*P* < 0.02). (**D**) ChIP-seq H3K9me3 profiles at selected loci in *Setdb1 tm1c/tm2* mutant ES cells at timepoints before and following a 48 h 4′OHT treatment. *T* = 0, untreated cells at start of timecourse; *T* = 2d/4d/6d, 2/4/6 days after start of 4′OHT treatment. (**E**) The percentage of reads present on endogenous retrovirus repeats (ERVs) or Alu repeats during the same timecourse as in (**D**), * indicates sample significantly different to *T* = 0 sample, *P* < 0.01 (Fisher’s Exact test) with the additional requirement of >1.5-fold change between the profiles.

To further gain insight into Setdb1 function, we assessed changes in gene expression by DNA microarray analysis and H3K9me3 chromatin marks genome-wide by ChIP-seq, over a 6 day 4′OHT timecourse of experimentally ablated and control *Setdb1* ES cells (Figure 3). Experimental and control mutant *Setdb1* ES cells were treated with 4′OHT for 48 h, while gene expression was analysed at days 0 (before treatment), 2 (end of treatment), 3, 4 and 6. Before 4′OHT treatment there were very few significant changes in gene expression between the experimental (*tm1c/tm2*) and control (*tm1c/^+^*) *Setdb1* ES cells, with only one gene, *Setdb1* itself, expressed at a lower level in the experimental cells as expected and 8 genes expressed at a higher level in the experimental cells (Supplementary Table S7). However, by day 2 after the start of 4′OHT treatment, there were 1331 genes showing significant changes in expression between the experimental null (*tm1d/tm2*) and control (*tm1d/^+^*) *Setdb1* ES cells, with roughly equal numbers of upregulated and downregulated genes. By day 6, 7848 genes had significant expression changes, with slightly higher number of genes downregulated as opposed to upregulated in the experimental (*tm1c/tm2*) null ES cells (*P* < 0.05; Supplementary Table S7).

We assessed changes in gene expression of pluripotency and differentiation markers (Figure 3A) and chromatin or epigenetic regulatory factors (Figure 3B). Many pluripotency factors and markers (e.g. *Nanog, Esrrb, Sox2, Pou5f1/Oct4*) show a gradual downregulation in the experimental null *Setdb1* ES cells compared to the control ES cells over time, and by day 6 most of these were significantly and strongly downregulated. Several differerentiation markers for multiple lineages including trophectoderm (e.g. *Cdx2*) were significantly strongly upregulated by the end of the timecourse. We also observed upregulation of some imprinted loci (e.g. *Mest, H19 and Igf2r*), suggesting that DNA methylation imprints are lost at these loci. Analysis of expression of chromatin factors (Figure 3B) also revealed significant changes, including in two DNA methyltransferases (*Dnmt1* and *Dnmt3b*) and other H3K9 methyltransferases (*G9a/Ehmt2/Bat8* and *GLP/Ehmt1)*, again occurring mainly towards the end of the timecourse. Consistent with our phenotypic observations, among the cohort of genes downregulated early on (in day 2 dataset) following *Setdb1* ablation were those associated with GO terms for stem cell maintenance, negative regulation of cell differentiation, regulation of embryonic development, and positive regulation of cell proliferation (Figure 3C). We identified a different set of enriched GO terms among the cohort of upregulated genes, notably relating to chromatin assembly, and chromosome organization or segregation. The GO terms of negative regulation of cell cycle and regulation of cell fate specification from this cohort are consistent with our phenotypic observation of reduced growth of ES cells following *Setdb1* ablation.

Setdb1 is known to trimethylate histone H3 on lysine 9 and this correlates with its repressive function using a reconstituted chromatin transcriptional activation system (55,56). Although we found no change in bulk H3K9me3 levels in *Setdb1* null ES cells (Supplementary Figure S5d), we looked for locus-specific alterations of H3K9me3 using ChIP coupled with massively parallel DNA sequencing (ChIP-seq) over a timecourse in 4′OHT treated *Setdb1* null ES cells versus control ES cells. We found progressive reductions in H3K9me3 at many known targets of Setdb1 (57–60), including imprinted genes (e.g. *H19*), olfactory receptors (e.g. *Olfr901*), trophoblast development factors (e.g. *Cdh3*) (Figure 3D and Supplementary Figure S6a). In our profiling, 67 and 86% respectively, of the Setdb1-regulated H3K9me3 sites previously identified by Bilodeau *et al.* (57) and Yuan *et al.* (60) using RNAi knockdown of *Setdb1* in mouse ES cell lines, show >1.5-fold reduction of H3K9me3 6 days after 4′OHT induction. Even by 2 days after induction, 35% of the H3K9me3-reduced sites identified by Yuan *et al.* (60) show H3K9me3 reduction in our experiments, identifying these regions as rapidly affected targets of Setdb1. We also observed a prominent loss of H3K9me3 over endogenous retroviral elements following *Setdb1* ablation (Figure 3E and Supplementary Figure S6b) and at additional gene loci (Supplementary Figure S6c). To take potential antibody crossreactivity into account, results were confirmed with three different H3K9me3 antibodies (Supplementary Figure S6a–c).

### Effect of enhancing pluripotency-promoting conditions on the growth of inducible conditional *Setdb1* mutant ES cell lines

**Figure 4.**
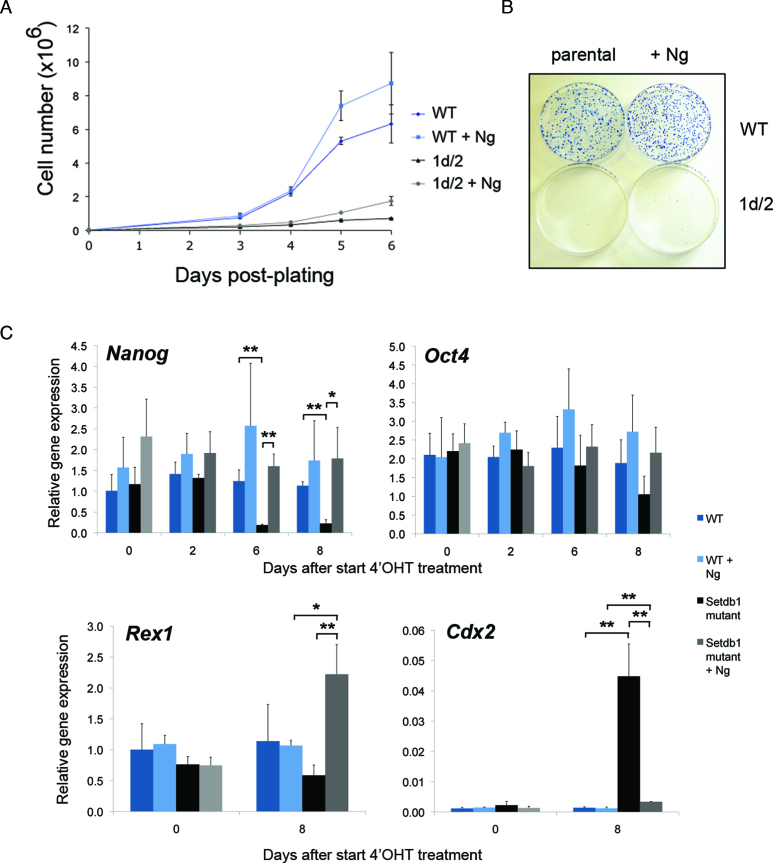
Effect of enhancing pluripotency-promoting conditions by constitutively expressing transgenic Nanog (+Ng) on inducible conditional Setdb1 mutant ES cell lines. (**A**) Growth of Setdb1 tm1d/tm2 mutant +Ng ES cells remains compromised, while control wild-type (WT) JM8 ES cells (without or +Ng) show robust growth characteristics. Cells were plated following a 48 h 4′OHT treatment and counted at the time intervals indicated. Results show the mean with error bars indicating the s.d. of three independent experiments. Data shown are from one representative cell line per genotype; similar results were obtained using three additional independent cell lines per genotype. (**B**) Setdb1 tm1d/tm2 mutant +Ng ES cells plated at low density remain unable to form normal sized colonies of undifferentiated ES cells, whereas 4′OHT treated control WT JM8 ES (without or +Ng) cells generate robust ES cell colonies. Cells were stained with methylene blue 8 days after plating on 10 cm tissue culture dishes. (**C**) Analysis of gene expression by quantitative RT-PCR at indicated timepoints before and following a 48 h 4′OHT treatment of Setdb1 tm1c/tm2 (becoming tm1d/tm2 after treatment) mutant ES cells (Setdb1 mutant) and control JM8 ES cells (WT), each +Ng, compared to the respective parental cell lines without the Nanog transgene. Results show mean ± s.d. of three independent experiments; asterisks indicate statistically significant differences between sample groups indicated with a bar (*, *P* < 0.025, **, *P* < 0.01; two-tailed Student’s *t*-test).

Given the striking decrease in expression of pluripotency associated genes and increase in differentiation associated genes following *Setdb1* depletion in ES cells, we sought to determine whether forced promotion of pluripotency conditions in the absence of *Setdb1* would rescue the growth reduction phenotype. To this end, we adopted two experimental approaches. The first was introducing a constitutively active *Nanog* (61) transgene (+Ng) into the genome of *Setdb1 tm1c/tm2* mutant ES cells and into JM8 WT ES cells as controls and monitoring growth and expression of key pluripotency and differentiation genes upon *Setdb1* ablation (Figure 4 and Supplementary Figure S7). We reconfirmed genotypes of clones with the *Nanog* transgene using our existing genomic PCR assays (Supplementary Figure S7a and b). Immunofluorescence microscopy showed that Nanog expression was maintained in the majority of +Ng *Setdb1 tm1d/tm2* null ES cells for at least 7 days after 4′OHT treatment of *Setdb1 tm1c/tm2* mutant ES cells, whereas in *Nanog*-untransfected parental *Setdb1 tm1d/tm2* null ES cells under the same treatment very few Nanog expressing cells were detectable, consistent with *Nanog* mRNA downregulation observed in our gene expression microarray timecourse analysis (Supplementary Figure S7c and Figure 3A). Enforced Nanog expression was unable to rescue the growth defect of *Setdb1 tm1d/tm2* null ES cells (Figure 4A) and these cells are unable to form ES cell colonies (Figure 4B), whereas control JM8 WT ES cells (with or without the *Nanog* transgene) cultured concurrently show robust growth characteristics. Strikingly, in +Ng *Setdb1 tm1d/tm2* null ES cells (post-4′OHT treatment), in which *Nanog* expression is restored as expected, expression of *Oct4* and *Rex1* returns to levels similar or higher than those in WT and derepression of *Cdx2* is substantially diminished (Figure 4C). As an independent test of enhancing pluripotency-promoting conditions on growth of *Setdb1* mutant ES cells, we cultured *Setdb1* mutant and control ES cells in LIF plus ground state pluripotency ‘two-inhibitor’ (2i) media (2,45) and reconfirmed clone genotypes by PCR (Supplementary Figure S8a). *Setdb1 tm1d/tm2* null mutant ES cells grown in 2i+LIF also exhibit a severely impaired growth rate similar to that in serum+LIF conditions (Supplementary Figure S8b) and cannot form undifferentiated ES cell colonies (Supplementary Figure S8c), despite having uniformly high levels of Nanog expression throughout the culture (Supplementary Figure S8d). We conclude that neither of the two naive pluripotency-promoting conditions (*Nanog* transgene expression or 2i+LIF culture) tested are able to rescue the severe self-renewal defect in ES cells upon *Setdb1* ablation.

## DISCUSSION

The modular design of IKMC Intermediate Vectors from the extensive KOMP-CSD and EUCOMM mouse knockout project resources (31,35) provides a flexible platform for engineering a wide variety of useful alleles using both replacement and insertional gene targeting strategies. Here we have utilized this resource to develop an insertion-type targeted trapping vector (pI_hygGFP) and use it to constitutively inactivate the second allele in existing IKMC heterozygous ‘knockout-first’ mutant mouse ES cells, thereby generating bi-allelic null ES cells for the gene of interest. Making a pI_hygGFP vector for a gene with an Intermediate Vector represented in the IKMC resource is amenable to non-specialists in vector design, since all of the modules required for building the final targeting vector already exist. The pI_hygGFP targeting vector is assembled *in vitro* in a three-way Gateway™ exchange reaction between the IKMC Intermediate Vector and two purpose built vector modules that we designed: an adaptor module (pL1L2_RloxP) and the appropriate reading frame version of the hygromycin-GFP gene trap vector (pL3L4_hygGFP). The pI_hygGFP vector is introduced, typically by electroporation, into an IKMC heterozygous ‘knockout-first conditional ready’ (containing the *tm1a* allele) mutant ES cell line, and clones are screened by PCR based genotyping to isolate bi-allelic null ES cell lines for the gene of interest. Our strategy is highly efficient for the generation of bi-allelic null mutant mouse ES cells, incorporates a GFP marker in the second (*tm2*) allele and can potentially be applied to thousands of mutant ES cells in the public IKMC resource from the KOMP-CSD and EUCOMM pipelines, containing heterozygous null mutations in expressed genes.

We observe a high average frequency of bi-allelic targeted clone recovery of 60% for 11 genes expressed in ES cells. A novel design feature of our ‘second allele’ targeting strategy is that the hygromycin selectable marker is not activated in clones that have re-targeted the ‘knockout-first’ (*tm1a*) allele (in *trans*), hence re-targeting of the existing ‘knockout-first’ mutant allele is selected against. The high targeting efficiency of our system reflects the combined effects of using: (i) insertion-type targeting vectors that are intrinsically more efficient than replacement-type vectors (4,62); (ii) ‘targeted trapping’ cassettes that enrich for correctly targeted events (10,35); and (iii) preferential recovery of second allele (in *cis*) targeting events by direct drug selection. Our method is also time efficient as the number of electroporated colonies required for screening to obtain the desired event is low; we routinely screened 24 clones per targeting experiment. Furthermore, this approach can be used to identify essential genes in ES cells. Provided that the targeting ability of the pI_hygGFP vector is validated in WT ES cells, the absence of colonies upon appropriate drug selection following targeting with the pI_hygGFP vector of IKMC heterozygous cells already containing the ‘knockout-first’ (*tm1a*) allele indicates that the gene is required for ES cell survival.

To enable further studies and extend the usefulness of resources generated by the IKMC project, we set out to investigate the function of chromatin or epigenetic factors expressed in ES cells as proof of principle of our bi-allelic gene targeting approach. ES cells have been successfully used as a model system to investigate chromatin structure and function (63–65). We generated bi-allelic mutant ES cell lines for 11 epigenetic regulators, and we identified three essential genes in ES cells: *Ddx27, Phf20* and *Setdb1*. Two of our bi-allelic mutant ES cell lines, for the *Jarid2* and *Jmjd2c* (also known as *Kdm4c*) genes, have been used in separate studies to further investigate regulation of epigenetic factors on pluripotency and differentiation (52,66).

For viable null mutant ES cells displaying a phenotype, the ability to perform genetic reversion experiments is the preferred method for establishing causality, as it offers a more precise and less invasive way to reinstate gene expression, due to the resulting minimal sequence changes introduced in the genome than BAC or cDNA-based transgenic approaches which may confound the rescue phenotype. Through the action of Flp recombinase we generated revertant ES cells from our viable *Jarid2* and *Cbx1* null *tm1a/tm2* mutant ES cells, confirmed reappearance of the full length protein, and investigated gene expression profiles by microarray in the null mutants compared to revertant ES cell lines. Cbx1 is one of three mammalian Heterochromatin Protein 1 homologs that bind pericentromeric and telomeric heterochromatin, and a different *Cbx1* constitutive knockout mouse model exhibited perinatal lethality due to a defect in neuromuscular development (67). However, consistent with a later study (68), there was no increased tendency of *Cbx1* null ES cells to differentiate during our routine cell culture. The paucity of gene expression changes upon reversion of our *Cbx1* null to heterozygous mutant ES cells is consistent with the absence of an overt phenotype in *Cbx1* null ES cells. Jarid2 belongs to the Jumonji histone lysine demethylase family and is a component of the PRC2 Polycomb repressive complex in ES cells which confers histone H3K27 trimethylation (69). In a separate study (52), our *Jarid2* null ES cells were shown to be severely compromised in multi-lineage differentiation and their ability to induce lineage-specific gene expression *in vitro* upon receipt of differentiation cues, concordant with a lack of recruitment of Polycomb PRC1 complexes and failure in establishing poised RNA Polymerase II at PRC2 target genes. The findings from our gene expression analyses comparing null to revertant *Jarid2* ES cells are consistent with previous studies comparing WT to *Jarid2* null mutant or RNAi knockdown ES cells (52,70–73), in that we identified aberrant expression of targets of Suz12 (a PRC2 component) and histone H3K27-trimethylated genes, as well as enrichment of GO categories relating to cell differentiation and developmental pathways and RNA polymerase II transcription factor activity. We demonstrated functional reversion of a molecular phenotype in our revertant *Jarid2 tm1c/tm2* ES cells, by showing restored recruitment of the Polycomb PRC1 component Mel18 at PRC2 target gene promoters, thereby confirming causality and providing a relevant example of the efficacy of our genetic reversion system.

During the course of our gene targeting experiments, we identified three putative essential genes in ES cells including *Setdb1*, through our inability to recover bi-allelic targeted clones while the individual pI_hygGFP vectors readily targeted WT ES cells. The flexibility of the IKMC allele structure allows for creation of inducible conditional mutant ES cells, which we demonstrated for *Setdb1* using our pI_hygGFP vector to constitutively inactivate one allele while conditionally inactivating the other allele, as further proof of principle of our system, and which allowed us to explore Setdb1 function in regulating pluripotency and self-renewal in detail. Setdb1 is a histone H3K9 methyltransferase that can act as a transcriptional repressor (55,56). Zygotic *Setdb1* ablation in mice leads to peri-implantation lethality and inability to derive *de novo* ES cells from inner cell mass cells (74). These observations are consistent with our inability to generate *Setdb1* constitutive null ES cells by sequential gene targeting of ES cells. The phenotype of our inducible conditional *Setdb1* null ES cells following 4′OHT treatment agrees well with published *Setdb1* shRNA knock down and conditional null ES cell studies, as we have shown promiscuous differentation, a severe growth defect, and locus-specific reductions in H3K9me3 marks at many Setdb1 target genes and some endogenous retrovirus elements (57–60,75–77). Our results strongly suggest that Setdb1 is required to maintain both self-renewal and the pluripotent state in ES cells.

Our approach enabled time-course studies of *Setdb1* gene ablation induced by 4′OHT treatment, minimized genetic differences between the control and experimental cell lines, while importantly also controlling for the effect of 4′OHT by treating both the control and experimental samples concurrently, advantages which are lacking in several earlier Setdb1 studies in ES cells (57,59,60). We observed progressively reduced expression of pluripotency factors, along with increased expression of differentiation markers of all three germ layers and also trophectoderm, germline, and imprinted genes, after *Setdb1* ablation over a 6 day timecourse, which broadly agrees with previous analyses (58,60). Our *Setdb1* ablation day 2 timecourse set of upregulated genes showed enrichment for GO terms relating to chromatin assembly and chromosome organization and segregation, consistent with previous work demonstrating Setdb1 requirement for proper spindle organization in oocyte maturation (78) and role in re-establishing pericentric heterochromatin during DNA replication (79). Previous reports (57–60) and our data suggests that repression of pluripotency gene pathways could lead to the growth and differentiation defects observed in *Setdb1* depleted ES cells. To address this issue, we chose to further apply our pI_hygGFP vector targeted inducible conditional mutant *Setdb1* ES cells to tease apart the roles of Setdb1 in regulating pluripotency, differentiation, and self-renewal, by experimentally enforcing ground state pluripotency on Setdb1 ablated ES cells and asking if this would rescue any aspect of the *Setdb1* ablation phenotype. We utilized two approaches; the first, to introduce a constitutively active *Nanog* transgene into the genome of *Setdb1* inducible mutant ES cells; the second, by culturing the cells in ‘two-inhibitor’ (2i) containing media, which captures ES cells in the ground state of pluripotency by suppressing the Gsk3 and Fgf-MAPK differentiation-promoting signalling cascades, leading to homogenous high levels of Nanog expression (2,45,61). Nanog is a key pluripotency regulator (61,80–84), the *Nanog* locus is a direct target of Setdb1 (57,60), ES cells are not overly sensitive to dosage-related differentiation induction from altered expression of Nanog compared to that of other key pluripotency factors (85,86) and *Nanog* was one of the most rapidly and strongly downregulated genes among the pluripotency factors upon *Setdb1* ablation in ES cells according to our gene expression timecourse analysis.

Interestingly, while Nanog mRNA and protein expression remained homogenous and at high levels as expected under both pluripotency-promoting conditions we tested, neither condition was able to rescue the severe self-renewal/growth defect upon *Setdb1* ablation in ES cells. Pluripotency is maintained by a genetic regulatory network centered around the core transcription factors Oct4, Sox2 and Nanog, which coordinately directly activate their own gene expression and repress activity of lineage-specific transcription factors, thereby suppressing differentiation (61,80). Oct4 acts in a mutually repressive direct regulatory feedback loop with Cdx2 to distinguish between embryonic and extraembryonic lineages (85,86). *Setdb1* depleted ES cells show an enhanced ability to differentiate along the trophoblast lineage and derepress the trophectoderm differentiation factor *Cdx2*, which has been identified as a direct target of Setdb1 by ChIP analysis (58–60). Previous studies suggested that Setdb1 maintains pluripotency in part through direct repression of *Cdx2* via interaction with Oct4 that is dependent on SUMOylation of Setdb1 and support a view that derepression of *Cdx2* in *Setdb1* depleted ES cells and blastocysts is directly due to a loss of Setdb1-dependent H3K9 methyation rather than a secondary consequence of Oct4 depletion (58–61,86). While our gene expression results confirm derepression of *Cdx2* in *Setdb1* ablated ES cells, we surprisingly found no evidence of altered H3K9me3 at the *Cdx2* locus in our ChIP-seq analyses. Enforcing constitutive expression of *Nanog* in the absence of *Setdb1* in ES cells led to restoration of gene expression of the pluripotency transcription factor *Oct4* and marker *Rex1*, and further led to a striking reduction of *Cdx2* derepression to near normal (low to absent) expression levels. These results suggest that (i) pluripotency can be rescued in the absence of *Setdb1* gene expression by over-riding Setdb1-mediated *Nanog* downregulation, and (ii) misregulation of *Cdx2*, and therefore restriction of trophoblast lineage potential in ES cells, is primarily influenced by direct loss of *Nanog* following *Setdb1* depletion rather than via direct regulation by Setdb1, as previously suggested (59). Collectively, our results strongly suggest that Setdb1 regulation of pluripotency is mediated primarily through its direct action in maintaining normal expression levels of Nanog. Setdb1 regulation of proliferation/self-renewal is likely mediated through a separate pathway than its regulation of pluripotency, rather than the reduction in growth rate being a secondary consequence of increased differentiation arising from exiting the pluripotent state following *Setdb1* depletion.

To assist users in successfully applying our bi-allelic targeting strategy to their gene(s) of interest, we discuss additional applications, potential caveats, and important quality control measures. We validated our second allele targeting strategy in IKMC *tm1a/^+^* ES cells generated with a ‘knockout-first’ promoterless vector. As an extension of applications using our pI_hygGFP vector, we note that other classes of heterozygous null mutant ES cells from the IKMC resource should be amenable to our targeting strategy to generate bi-allelic null ES cells, provided that the gene is expressed at a sufficiently high level to enable effective hygromycin selection. All KOMP-CSD and EUCOMM ‘knockout-first’ vectors, both promoterless and promoter-driven, were constructed from Intermediate Vectors of the same format, it is the Gateway-adapted plasmids used for the final assembly step in the high-throughput vector construction pipeline which differ (35), and hence it is possible to construct a pI_hygGFP insertion vector for any Intermediate Vector. In total, there are 16 296 vector designs for first allele targeting (including promoterless and promoter-driven vectors) representing 15 185 different genes within the IKMC resource, collectively produced by the KOMP, EUCOMM and NorCOMM projects (http://www.mousephenotype.org/about-ikmc); this resource contains ES cells with mutant alleles for expressed and non-expressed genes, and a variety of first mutant allele types (31,33,35). Expressed genes represented by heterozygous mutant ES cells containing the EUCOMM or KOMP-CSD ‘promoter-driven selection cassette’ class *tm1a* allele, *tm1e/+* ‘targeted non-conditional’ allele containing ES cells, and *tm1/^+^* or *tm1.1/^+^* ‘targeted deletion’ ES cells, should be amenable to bi-allelic targeting with a pI_hygGFP vector. Our strategy will not work in conjuction with designs from the KOMP-Regeneron project as they utilized a different vector construction pipeline in which there are no Intermediate Vectors (31). Detailed information on all IKMC produced vectors and alleles are available through the IKMC Targeting Repository at the IMPC web portal (33) (https://www.mousephenotype.org/imits/targ_rep).

We expect that a threshold of gene expression level will exist to permit successful targeted trapping using our pI_hygGFP vector. Although we have not quantified the lower threshold, a separate study using different targeted trapping vectors was able to define a minimum threshold for success as 1% of the expression level of the ‘housekeeping’ transferrin receptor (*Trfr*) gene, and reported a high success rate for genes expressed at >5% of *Trfr* (10). Another indicator of high probability for success of pI_hygGFP vector targeted trapping into other classes of heterozygous null mutant ES cells from the IKMC projects is that the gene of interest has previously been gene trapped, which can be checked against the International Gene Trap Consortium database (7) (http://www.genetrap.org/). We advise researchers interested in applying our bi-allelic targeting method to classes of first mutant alleles other than those made with IKMC promoterless vectors to confirm that their gene of interest is expressed above 1% *Trfr* level in ES cells and/or has been gene trapped, prior to ordering the relevant Intermediate Vector from the IMPC for pI_hygGFP vector construction.

Our approach assumes that the IKMC *tm1a* alleles (31,35) and *tm2* alleles are null alleles. The EUMODIC and Sanger Institute MGP large-scale mouse phenotyping pilot projects successfully used the *tm1a* allele to generate over 600 mutant mouse lines and noted that this design gave rise to null alleles for most genes (38,39,87). However in some cases, failure of complete transcription stop at the inserted vector cassette occurred, in which case the *tm1a* allele led to a knockdown of gene expression (87). Recognizing this, subsequent and ongoing phenotyping programmes within the IMPC are producing mutant mice from the Cre-recombined *tm1b* allele, in which removing a ‘critical exon’ is designed to induce a frame-shift mutation (35), as an extra precaution to increase the probability of generating a null allele (40,41,88). Should the *tm1a* allele for an ES cell expressed gene of interest prove to be inadequate, our strategy for second allele targeting with the pI_hygGFP vector can also be applied to *tm1b/^+^* ES cells; however, the *tm1b* null allele is not revertible nor drug-selectable. The design of the *tm2* allele, as for the *tm1a* allele, relies on an introduced stop codon in the trapping cassette to terminate transcription, thus there is a risk that splicing over or incomplete termination could occur. As with any gene modification approach, researchers must carefully validate each allele and mutant ES cell line prior to use in functional studies.

The EUCOMM and KOMP-CSD vector construction and gene targeting pipelines were high-throughput, and therefore ES cell clones from the resource were genotyped for correct targeting by PCR only (35). Analysis of 9496 IKMC vector gene targeting events revealed that the proportion of clones with random integrations was 2.1% (25). Given the possibility of complex vector integration events or random integrations, we recommend that researchers use additional methods (e.g. Southern blot with an external probe) to verify proper targeting of the intended allele prior to extensive use of bi-allelic mutant ES cells in functional studies. To detect the presence of random vector integrations into the genome, Southern blot with a probe from the selectable marker or vector cassette, or methods to measure copy number variation such as quantitative PCR (e.g. droplet digital PCR), could be applied. Ryder *et al.* (38) found that of 731 EUCOMM or KOMP-CSD ES cell clones achieving germline transmission, 86% passed quality control (QC) testing and the failures were typically due to having mixed ES cell clones. Mixed clones can readily be resolved through subcloning and re-screening a number of subcloned lines.

We recommend that researchers perform QC checks on control and mutant ES cells, including assessing chromosome integrity and pluripotency status. ES cells should ideally be QC checked throughout their period in culture, as abnormalities could develop over extended time in culture. Chromosome integrity can be monitored by karyotyping or analysis of copy number variation. Standard indicators of pluripotency of mouse ES cell lines include positive staining for AP activity and expression of core and naïve pluripotency markers including Oct4, Sox2, Nanog, Esrrb, Klf2/4 and Tfcp2l1 (2). The defining test of pluripotency is the ability of the cell to contribute to all developmental lineages in a chimeric embryo following introduction into a host blastocyst and be germline competent. Germline transmission rates of EUCOMM vector targeted JM8, JM8.N4 and JM8.F6 mouse ES cell clones ranged from 62–69% (44) and current germline transmission rates for targeted KOMP-CSD ES clones in these same genetic backgrounds range from 59 to 66% (https://www.komp.org/gltrates2.php). Prior to ordering clones, we recommend checking the IMPC web portal (https://www.mousephenotype.org) to determine whether the researcher’s allele of interest has undergone germline transmission and passed IMPC QC screening, in order to request the highest quality clones. For alleles with no germline transmission data, we recommend that users QC test up to three ES cell clones (*tm1a/^+^*) from IKMC/IMPC repositories in order to obtain at least one that passes QC for use in bi-allelic targeting. For the phenotyping experiments performed in this study we used a minimum of three independent clones of each genotype (biological replicates) of bi-allelic targeted ES cells, rather than relying on technical replicates. In this way, stringency is increased and the impact of any one clone having off-target or other genetic defects is minimized. However the final choice of the number of independent clones used will be driven by the research hypothesis, study design, and statistical methods employed.

In summary, our work demonstrates the feasibility of performing gene ablation and genetic reversion experiments, using existing vectors and gene targeted heterozygous ‘knockout-first’ *tm1a/^+^* mutant mouse ES cells which are readily available from IKMC/IMPC public resources, combined with our new insertional targeted trapping pI_hygGFP vectors for bi-allelic gene targeting of expressed genes to rapidly and efficiently generate null ES cells. Our approach was applied as proof of principle to investigate the function of chromatin and epigenetic regulators including *Jarid2* and *Cbx1*, and can be applied to identify genes essential for basic cellular and developmental processes using mouse ES cells. Application of our technology for the generation of inducible conditional mutant ES cell lines, such as we have illustrated for *Setdb1*, will facilitate further investigations of essential genes in both undifferentiated ES cells and *in vitro* cell differentiation models. Rapid production of bi-allelic null mouse ES cells using our method, which builds upon existing and extensive IKMC resources, will enable their use in a wide range of gene function studies, validating and complementing current approaches using genome-editing technology which has a higher propensity of off-target effects. Our method for bi-allelic targeting maximizes usage of cost-efficient readily available IKMC resources and will further enable the use of mouse ES cells as a model system for fundamental investigations of pluripotency and differentiation pathways and studies in regenerative medicine.

## DATA AVAILABILITY

Information on all available Intermediate Vectors and targeted ES cells from the EUCOMM and KOMP-CSD mutant mouse resource projects generated by the IKMC can be obtained via the IMPC website at http://www.mousephenotype.org/. IKMC Intermediate Vectors, ‘knockout-first, conditional ready’ and other IKMC final targeting vectors, JM8 WT and heterozygous ‘knockout-first, conditional-ready’ (*tm1a*/^+^) mutant ES cells can be ordered from IMPC public repositories via the IMPC web portal (http://www.mousephenotype.org). Plasmids generated in this study will be available from Addgene (https://www.addgene.org/).

Microarray gene expression data have been deposited in the ArrayExpress database at EMBL-EBI (www.ebi.ac.uk/arrayexpress) under accession numbers E-MTAB-5930 for *Jarid2* and *Cbx1*, and E-MTAB-5931 for *Setdb1* mutant ES cell experiments respectively. All ChIP-seq data (FASTQ, BED and WIG files) are present in the NCBI GEO SuperSeries GSE31777.

## Supplementary Material

Supplementary Data
