# Supplementary material for: An efficient method for generation of bi-allelic null mutant mouse embryonic stem cells and its application for investigating epigenetic modifiers
Source: Nucleic Acids Res. 2017 Sep 13;45(21):e174. doi: 10.1093/nar/gkx811 (PMC5716182; doi:10.1093/nar/gkx811)
Supplement: Supplementary Data [file gkx811_supp.zip › Fisher_SUPPL_MandM_gkx811_040917.pdf]

SUPPLEMENTARY MATERIALS AND METHODS

Cell based assays and microscopy

ES cells plated either under normal monolayer conditions or grown as colonies were fixed and stained with X-Gal as described (1,2) to detect betagalactosidase expression from the lacZ-neo (betageo) targeting cassette, present in the *tm1a* mutant allele but not in the *tm1c*, *tm1d*, or *tm2* alleles. Cells expressing betagalactosidase stain blue whereas non-expressing cells remain unstained (**Supplementary Figure S2**).

To confirm GFP expression from the hygromycin-GFP cassette in the *Setdb1 tm2* mutant allele that generated a stable truncation product (**Figure 2**), FACS analysis on unstained live cells, and immunofluorescence microscopy on fixed cells using an antibody against GFP were used (**Supplementary Figure S5**). For FACS, cells were washed and resuspended in PBS with 1% BSA, and analyzed on a FACS Calibur flow cytometer. Data was analyzed with CellQuest software from Becton Dickinson. For microscopy, ES cells were plated onto glass coverslips in 6 well plates coated with 0.1% gelatin, and were fixed two to 24 hours later in 2% paraformaldehyde in PBS for 20 minutes at room temperature, washed several times in PBS and stored at 4°C under PBS for up to two months prior to antibody incubation. Cells on coverslips were permeabilized with 0.4% Triton X-100 in PBS for 5 minutes, blocked in 2.5% BSA, 0.05% Tween-20, 10% goat serum in PBS, followed by incubation with either anti-GFP antibody (Invitrogen, A11122) at 1:5000 or anti-Nanog antibody (Cosmo-Bio Co. Ltd, RCAB0002P-F) at 1:200 and Alexa Fluor 488 or 568 conjugated IgG secondary antibodies (Invitrogen, A11034, A11011) at 1:400, stained and mounted onto microscope slides using Vectashield containing 1.5 μg/ml DAPI (Vector Labs). Cells were imaged using a Leica SP5 confocal microscope with the 405nm Diode laser to detect the DAPI signal, an Argon laser for detection at 488nm, and a DPSS 561nm laser for detection at 568nm. LAS-AF software was used for image acquisition and TIFF files were exported into Adobe Photoshop to prepare final images. The contrast was manually adjusted by an equal amount in all GFP images presented to better visualize the GFP signal.

To detect alkaline phosphatase activity in ES cells, the 86-R (Sigma) kit with the FRV-Alkaline and Hematoxylin Gill no. 3 solutions, was used according to manufacturers instructions with modifications to cell fixation conditions as described (2).

Colony assays were performed under routine culture conditions, using ES cells after an initial 48 hour treatment period with 4'OHT plus variable length additional culture time without 4'OHT (as indicated for each experiment, from 0-4 days) by plating an equal number of cells at low density (1 or 2 x 10^3, as indicated for each experiment) of identically treated *Setdb1* mutant and control ES cells onto separate 10cm diameter gelatinized tissue culture dishes. After 8-12 days of growth the cells were fixed and stained with methylene blue (Stemcell Technologies) to visualize relative colony abundance and morphology. Colony assays were performed in triplicate and representative examples are shown. Growth curves were measured by plating 2 x 10^4 each of *Setdb1* mutant or control ES cells following an initial 4'OHT treatment and culture period as above, in triplicate 6-well gelatinized plates, then harvested at subsequent time intervals by trypsinization and live cell numbers were counted using a hemocytometer and Trypan Blue staining (Stemcell Technologies).

ES cells were imaged directly on the culture plate following staining for alkaline phosphatase, X-Gal, or methylene blue, using an Olympus IX51 compound light microscope with the 10x objective lens (or the 4x objective for the *Setdb1* ES cell individual colony images) with the ColorView camera system and DP-Soft image capture software. The 10cm colony assay plates were imaged using a hand-held Lumix DMC-TZ5 digital camera. Images were exported into Adobe Photoshop for final figure preparation with no image manipulation.

Western blot analysis

Nuclear extracts were prepared from ES cells plated to 70-90% confluence on 10cm dishes by washing once in ice cold PBS then incubating over ice for 1-5 minutes in 1mL ice cold harvesting buffer (2.5 mM $MgCl_2$, 0.5% IGEPAL CA-630 (Sigma I8896), 10mM Sodium phosphate buffer at pH7.2) containing 0.5mM DTT and 1:200 Protease Inhibitor cocktail (Sigma P8340). Samples were recovered using a cell scraper, placed into a 1.5 mL tube and centrifuged 2 min at 3000 rpm at 4^oC. The pellet was resuspended in 200 μl ice cold RIPA buffer (150mM NaCl, 0.5% IGEPAL CA-630, 0.5% deoxycholate, 0.1% SDS, 50mM Tris-HCl pH 8.0) containing 2mM DTT and 1:100 Protease Inhibitor cocktail, and was syringe passaged with a 1ml syringe 5 times with a 20 gauge needle and then 5 times with a 25 gauge needle, over ice. Nuclear extracts were subjected to SDS-PAGE using the Mini-Protean 3 Cell (BioRad) and immediately prior to loading onto Ready Gel Tris-HCl gels (BioRad), samples were mixed with 4X LDS sample buffer (Invitrogen) pre-warmed to 90^oC, and were heated for 10-15 minutes at 95^oC. 10-20 μg of nuclear extract was loaded per well and 10 μL of Novex Sharp Protein Standard (Invitrogen LC5800) was used for size markers. Gels were transferred onto Immobilion-P (Millipore) PVDF membranes, blocked at least 1 hour in 5% milk in TBS+0.1% Tween-20 (BioRad), then incubated with primary and HRP-conjugated secondary antibodies. Blots were incubated with primary antibodies against Cbx1 (Abcam 40828) at 1:10,000, Histone H3K9me3 (Abcam 8898) at 1:10,000, Oct4 (Santa Cruz 8628) at 1:5000, Jarid2 (Abcam 48137) at 1:10,000, Setdb1 (Millipore/Upstate 07-378) at 1:10,000, and alpha-Tubulin (Abcam 4074) at 1:10,000 dilutions, and secondary antibodies used were anti-goat IgG (Abcam 6741) or anti-rabbit IgG (Abcam 6721) each at 1:50,000 to 1:100,000 dilution. Products were detected by chemiluminescence using the Amersham ECL Advance detection kit (GE Healthcare) and imaged on Amersham Hyperfilm ECL (GE Healthcare).

Gene expression analysis

RNA was prepared from confluent ES cells on 12-well (for *Cbx1* and *Jarid2* samples) or 6-well (for *Setdb1* samples) plates for expression microarray analysis following trypsinization, one PBS wash, then cells were collected by centrifugation and lysed in 1ml Trizol (Invitrogen) and processed according to manufacturer's instructions. Total RNA samples were cleaned up using the RNeasy kit (Qiagen), quantitated using a nanodrop spectrophotometer, and RNA integrity was confirmed by running an aliquot on a 1% agarose-TAE gel. RNA samples were diluted to 50ng/μl in RNAse/DNase free water (Gibco), and 500ng total RNA per sample was subjected to cDNA synthesis and T7-directed RNA amplification using the Illumina™ TotalPrep™-96 RNA Amplification Kit (Ambion). Amplified and biotinylated cRNA (1500ng per sample) was hybridized to the Mouse WG-6 v2.0 BeadChip (Illumina) microarray for 20 h. Following hybridization, BeadChips were washed, and annealed probes were detected using Streptavidin-Cy3. All hybridisation and detection steps were carried out according to the manufacturer's instructions. Processed BeadChips were scanned using the Illumina BeadArray reader and annotated probe intensity values were extracted using Genome Studio software version 1.5.4 (Illumina). Array content was MouseWG-6_V2_0_R3_11278593_A (Illumina). Three (six for the *Cbx1* dataset) biological replicates (independent ES cell clones of the same genotype) were used to generate statistically significant gene lists. The array probe summaries were calculated in BeadStudio and were quantile normalised (3). Data were analyzed using the Bioconductor Lumi (4) and Limma (5) packages. A linear model fit was applied using Limma, data were p-value adjusted to yield a sorted list of differentially expressed genes using the Benjamini and Hochberg method (6). Microarray gene expression data have been deposited in the ArrayExpress database at EMBL-EBI (www.ebi.ac.uk/arrayexpress) under accession

numbers E-MTAB-5930 for *Jarid2* and *Cbx1*, and E-MTAB-5931 for *Setdb1* mutant ES cell experiments respectively.

For the Gene Ontology (GO) analysis, multiple probes corresponding to the same genes were filtered to the probes with the highest intensities, prior to enrichment testing. Genes found to be significant at a p-value of less than 0.05 were first grouped into their direction of change between conditions. These gene groups were then used for functional GO category enrichment using the hypergeometric test implemented in the topGO Bioconductor package (7) and with genome wide annotation for the mouse genome from the Bioconductor package org.Mm.eg.db.

Statistical analysis of changes in expression for transcription factor target gene sets in mutant ES cells was performed as follows. P-values were determined by Student's t-test for differential expression of transcription factor target genes (as defined by previous ChIP-seq studies listed below) versus global differential expression. Numbers of genes in each set are as follows: 1. Nanog: 1233, Oct4: 753, Sox2: 786, Klf4: 1702, Dax1: 1692, Nac1: 769, Rex1: 1480, Myc: 3414, Zfp281: 578, (8); 2. Nanog: 399, Oct4: 815, Sox2: 177, Klf4: 3677, Esrrb: 2703, Zfx: 4928, E2f1: 8052, Tfcp2l1: 3656, Smad1: 0, Stat3: 403, c-Myc: 2260, n-Myc: 4244, Suz12: 1390, CTCF: 1568 (9); 3. Zfp281: 2395 (10); 4. Sall4: 1065 (11), 5. Trim28: 3072, Cnot3: 1546 (12); 6. Suz12: 6030, Eed: 5590, Phc1: 5681, Rnf2: 5978, H3K27me3: 7316 (13).

For experiments involving the Nanog transgenic - inducible conditional *Setdb1* mutant ES cells, gene expression analysis was performed by real time quantitative PCR. RNA was harvested from ES cells directly in the culture plate using QIAshredder followed by extraction with the RNAeasy kit (Qiagen). 2 μg of total RNA was reverse transcribed using 200U SuperScriptIII with 300ng random primers as recommended by the manufacturer (Invitrogen). cDNA was resuspended in 100μL water, and quantification of 2 μL precipitated DNA per reaction was performed by real-time PCR amplification using Quantitect SYBR Green PCR mix (Qiagen) in a 20 μL reaction volume with 10 μM primers, on a Chromo4 DNA Engine thermal cycler (Biorad) running Opticon Monitor 3 software and using the following program: 95°C for 15s then 40 cycles of 94°C for 20s, 55°C for 20s, 72°C for 30s, followed by plate read. *Nanog*, *Oct4*, and *Rex1* primer sequences are as described (14,15), and gene expression was normalized against the average expression of the *Hmbs* and *Ywhaz* housekeeping genes as described previously (14,15); *Cdx2* primers are as described (16). Controls without reverse transcriptase were processed concurrently due to elimination of a DNase treatment step to avoid degradation during RNA processing, and were negative for target amplification.

Chromatin immunoprecipitation (ChIP)

ChIP-qPCR experiments on *Jarid2* null and revertant ES cells (on n=3 independent clonal cell lines per genotype) for Mel18 enrichment at known binding regions were performed as described (15) with modifications. Four 15cm dishes of 80-90% confluent ES cells per sample were crosslinked in situ with 1% paraformaldehyde for 10 min at 37°C, followed by quenching with 125mM Glycine for 5-20 min at room temperature, two washes in PBS at 4°C, and incubation in swelling buffer (25mM HEPES pH 7.9, 1.5 mM $MgCl_2$, 10 mM KCl, and 0.1% NP-40 and protease inhibitor cocktail (Roche)) for 10-30 min at 4°C. Cells were harvested by scraping, homogenized with 50 strokes in a Dounce homogenizer with tight pestle, centrifuged, and the pellet was sonicated in sonication buffer (50mM HEPES pH 7.9, 140mM NaCl, 1mM EDTA, 1% Triton X-100, 0.1% Na-deoxycholate, 0.1% SDS, and protease inhibitor cocktail (Roche) using a Bioruptor device (Diagenode). Fragmented pre-cleared chromatin (450 μg) was subjected to immunoprecipitation with 10 μg of anti-Mel18 (Santa Cruz sc-10744 H-115), or negative control anti-IgG-mix (Dako Z0259), and ProteinG-Dynabeads (Invitrogen). After elution of immune complexes, DNA was resuspended in 50 μL TE pH 8, and quantification of 2 μL precipitated DNA per reaction was performed by real-time qPCR amplification using Quantitect

SYBR Green PCR mix (Qiagen) in a 30 μL reaction volume with 10 mM primers, on a Chromo4 thermal cycler (Biorad) running the following program: 95°C for 15s then 40 cycles of 94°C for 20s, 55°C for 20s, 72°C for 30s, followed by plate read. Primer sequences are as described (15). The amount of DNA precipitated by each antibody was normalised against 10% (45 μg) of the starting input material.

ChIP-seq experiments were performed on *Setdb1* depleted and control ES cells using $3.3x10^6$ cells as described (17) with minor modifications. Crosslinking was performed on the culture plates for 10 minutes and immunoprecipitated DNA was purified using the QIAquick PCR purification Kit (Qiagen). ChIP enrichment levels were analyzed by qPCR for quality control. Antibodies and the amount used for ChIP are described in **Supplementary Table S8**.

Sequencing

ChIP DNA samples for the *Setdb1* depleted and control ES cells were prepared for sequencing by end repair of 20ng DNA as measured by Qubit (Invitrogen). Adaptors were ligated to DNA fragments, followed by size selection (~300bp) and limited PCR amplification (14 cycles). Quality control was performed by qPCR and running the products on a Bioanalyzer (BioRad). Cluster generation and sequencing-by-synthesis (36bp) was performed using the Illumina Genome Analyzer IIx (GAIIx) platform according to standard protocols (Illumina). Samples were sequenced to a depth of approximately 10-20 million mapped tags per sample. Sequences were aligned to the reference genome using the Illumina Analysis Pipeline allowing one mismatch. Only the tags uniquely aligning to the genome were considered for further analysis. For ChIP-seq identical sequence tags were discarded to obtain a non-redundant set, and the 36 bp sequence reads were directionally extended to 300 bp, corresponding to the length of the original fragments used for sequencing. The output data were converted to Browser Extensible Data (BED) files for downstream analysis and Wiggle (WIG) files for viewing. All sequencing analyses were conducted based on the Mus musculus NCBI m37 genome assembly (MM9) accessed from the UCSC Genome Browser (assembly July 2007). The sequencing output is summarized in **Supplementary Table S8**. All ChIP-seq data (FASTQ, BED and WIG files) are present in the NCBI GEO SuperSeries GSE31777.

ChIP-seq analyses

To compensate for differences in sequencing depth and mapping efficiency, the total number of unique tags for each sample (*Setdb1* depleted and control ES cells) was uniformly equalized, allowing quantitative comparisons (**Supplementary Table S8**). Examples (shown in **Figure 3** and **Supplementary Figure S6**) were selected based on known target sites of Setdb1. However, all examples are robustly called as enriched in our wildtype (T=0) profiles based on Poisson distribution probability, as generally recommended in the field (18).

To rule out the possibility of nonspecific signals from the H3K9me3 antibody (gift from Thomas Jenuwein; Millipore/Upstate 07-442-4861) used in our ChIP-seq timecourse experiments (**Figure 5**), we confirmed these observations with two additional H3K9me3 antibodies (**Supplementary Table S8 and Supplementary Figure S6**). For the profiles generated with the Abcam Ab 8898-58206 and the Upstate 07-442-4861 antibodies, we detected additional signals over promoters and gene bodies of active genes, which were absent in the profiles generated with the Abcam Ab 8898-638466 antibody (refer to GEO SuperSeries GSE31777) and may represent cross-reactivity. For the repeat analysis, mappings were performed using the maq (m̲apping and a̲ssembly with q̲ualities) aligner version 0.7.1 (19). The major advantage with maq as compared to the ELAND pipeline is that, if a sequenced read aligns on multiple places on the genome, maq places it at one random position. This is useful

when studying repeat classes, as the reads representing these classes will by definition map on multiple genomic locations, which however all belong to the same class of repeat. All reads mapped by maq were included in downstream analyses. To enable direct comparisons, the samples were ratio normalized for the total number of tags mapped by maq. Sequence coordinates of various repeat classes were downloaded from the UCSC Table Browser (RepMask 3.2.7; rmskRM327). ChIP-seq tags were considered to represent a repeat class in case of any overlap of the 36nt sequenced fragment with the repeat class. Subsequently, the number of ChIP-seq tags representing a repeat class was counted. Statistical differences between coverage of repeat elements in ChIP-Seq were determined using Fisher's Exact test ($p<0.01$) with the additional requirement of >1.5 fold change between the profiles.

REFERENCES

1. Skarnes, W.C. (2000) Gene trapping methods for the identification and functional analysis of cell surface proteins in mice. *Methods Enzymol*, **328**, 592-615.
2. Rathjen, J., Lake, J.A., Bettess, M.D., Washington, J.M., Chapman, G. and Rathjen, P.D. (1999) Formation of a primitive ectoderm like cell population, EPL cells, from ES cells in response to biologically derived factors. *J Cell Sci*, **112 (Pt 5)**, 601-612.
3. Yang, Y.H., Dudoit, S., Luu, P., Lin, D.M., Peng, V., Ngai, J. and Speed, T.P. (2002) Normalization for cDNA microarray data: a robust composite method addressing single and multiple slide systematic variation. *Nucleic Acids Res*, **30**, e15.
4. Du, P., Kibbe, W.A. and Lin, S.M. (2008) lumi: a pipeline for processing Illumina microarray. *Bioinformatics*, **24**, 1547-1548.
5. Smyth, G.K. (2004) Linear models and empirical bayes methods for assessing differential expression in microarray experiments. *Stat Appl Genet Mol Biol*, **3**, Article3.
6. Benjamini, Y. and Hochbert, Y. (1995) Controlling the false discovery rate: a practical and powerful approach to multiple testing. *Journal of the Royal Statistical Society. Series B (Methodological)*, **57**, 289-300.
7. Alexa, A., Rahnenfuhrer, J. and Lengauer, T. (2006) Improved scoring of functional groups from gene expression data by decorrelating GO graph structure. *Bioinformatics*, **22**, 1600-1607.
8. Kim, J., Chu, J., Shen, X., Wang, J. and Orkin, S.H. (2008) An extended transcriptional network for pluripotency of embryonic stem cells. *Cell*, **132**, 1049-1061.
9. Chen, X., Xu, H., Yuan, P., Fang, F., Huss, M., Vega, V.B., Wong, E., Orlov, Y.L., Zhang, W., Jiang, J. *et al.* (2008) Integration of external signaling pathways with the core transcriptional network in embryonic stem cells. *Cell*, **133**, 1106-1117.
10. Wang, Z.X., Teh, C.H., Chan, C.M., Chu, C., Rossbach, M., Kunarso, G., Allapitchay, T.B., Wong, K.Y. and Stanton, L.W. (2008) The transcription factor Zfp281 controls embryonic stem cell pluripotency by direct activation and repression of target genes. *Stem Cells*, **26**, 2791-2799.
11. Lim, C.Y., Tam, W.L., Zhang, J., Ang, H.S., Jia, H., Lipovich, L., Ng, H.H., Wei, C.L., Sung, W.K., Robson, P. *et al.* (2008) Sall4 regulates distinct transcription circuitries in different blastocyst-derived stem cell lineages. *Cell Stem Cell*, **3**, 543-554.
12. Hu, G., Kim, J., Xu, Q., Leng, Y., Orkin, S.H. and Elledge, S.J. (2009) A genome-wide RNAi screen identifies a new transcriptional module required for self-renewal. *Genes Dev*, **23**, 837-848.
13. Boyer, L.A., Plath, K., Zeitlinger, J., Brambrink, T., Medeiros, L.A., Lee, T.I., Levine, S.S., Wernig, M., Tajonar, A., Ray, M.K. *et al.* (2006) Polycomb complexes repress developmental regulators in murine embryonic stem cells. *Nature*, **441**, 349-353.

14. Azuara, V., Perry, P., Sauer, S., Spivakov, M., Jorgensen, H.F., John, R.M., Gouti, M., Casanova, M., Warnes, G., Merkenschlager, M. *et al.* (2006) Chromatin signatures of pluripotent cell lines. *Nat Cell Biol*, **8**, 532-538.
15. Landeira, D., Sauer, S., Poot, R., Dvorkina, M., Mazzarella, L., Jorgensen, H.F., Pereira, C.F., Leleu, M., Piccolo, F.M., Spivakov, M. *et al.* (2010) Jarid2 is a PRC2 component in embryonic stem cells required for multi-lineage differentiation and recruitment of PRC1 and RNA Polymerase II to developmental regulators. *Nat Cell Biol*, **12**, 618-624.
16. Niwa, H., Toyooka, Y., Shimosato, D., Strumpf, D., Takahashi, K., Yagi, R. and Rossant, J. (2005) Interaction between Oct3/4 and Cdx2 determines trophectoderm differentiation. *Cell*, **123**, 917-929.
17. Marks, H., Chow, J.C., Denissov, S., Francoijs, K.J., Brockdorff, N., Heard, E. and Stunnenberg, H.G. (2009) High-resolution analysis of epigenetic changes associated with X inactivation. *Genome Res*, **19**, 1361-1373.
18. Pepke, S., Wold, B. and Mortazavi, A. (2009) Computation for ChIP-seq and RNA-seq studies. *Nat Methods*, **6**, S22-32.
19. Li, H., Ruan, J. and Durbin, R. (2008) Mapping short DNA sequencing reads and calling variants using mapping quality scores. *Genome Res*, **18**, 1851-1858.
