# Supplementary material for: An efficient method for generation of bi-allelic null mutant mouse embryonic stem cells and its application for investigating epigenetic modifiers
Source: Nucleic Acids Res. 2017 Sep 13;45(21):e174. doi: 10.1093/nar/gkx811 (PMC5716182; doi:10.1093/nar/gkx811)
Supplement: Supplementary Data [file gkx811_supp.zip › nar-00912-met-g-2017-File006.pdf]

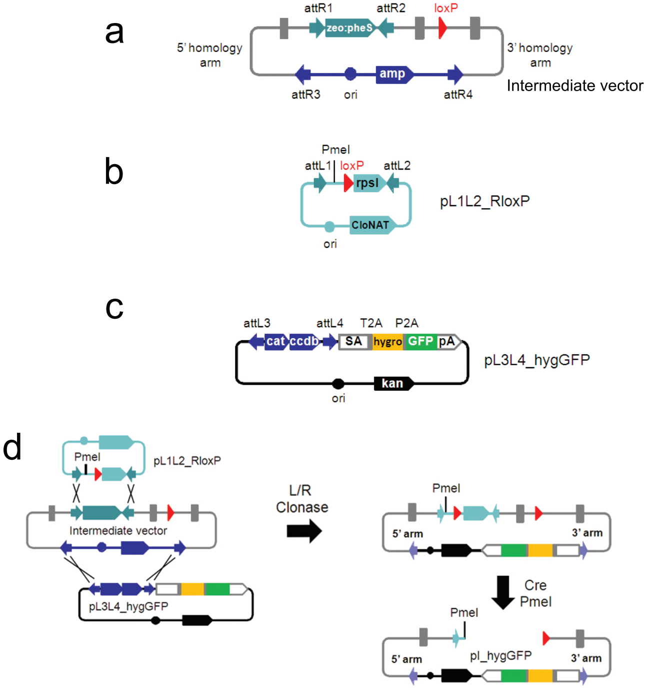

Supplementary Figure S1

Supplementary Figure S1. Assembly of insertion vectors by a multi-Gateway reaction used for targeted gene trapping of the second allele in IKMC heterozygous ‘knockout-first’ mutant embryonic stem cell lines.

(a) Schematic showing the structure of the Gateway-adapted Intermediate Vector from the IKMC resource. This vector contains the 5’ and 3’ genomic homology arms necessary for targeting by homologous recombination in ES cells, and a single loxP site, which will be used to create a Cre-mediated deletion (approximately 600 bp) of the floxed genomic region in *E. coli* in the final targeting vector to facilitate PCR-based genotyping of targeted ES cells.

(b) The L1/L2 Gateway-adapted loxP rpsl module (pL1L2_RloxP), which includes the rare restriction enzyme recognition site for PmeI for linearizing the final insertion targeting vector within the region of genomic homology, prior to electroporation of ES cells, and introduces a second loxP site, is used to create the deletion within the final targeting vector. The rpsl gene acts as a negative selection for subsequent Cre meditated deletion.

(c) The L3/L4 trapping cassette (pL3L4_hygGFP) contains a splice acceptor site (SA), a hygromycin resistance gene (hygro; in yellow), and an enhanced GFP marker (GFP, in green), separated by viral T2A and P2A ribosomal skipping elements respectively, followed by a poly-adenylation (pA) signal sequence for transcript termination.

(d) Diagram of the multi-plasmid Gateway reaction with the three vector components used to assemble the final, reading-frame specific vector, and schematic of the resulting insertion targeting vector (pI_hygGFP) following Cre-mediated recombination and linearization by PmeI.

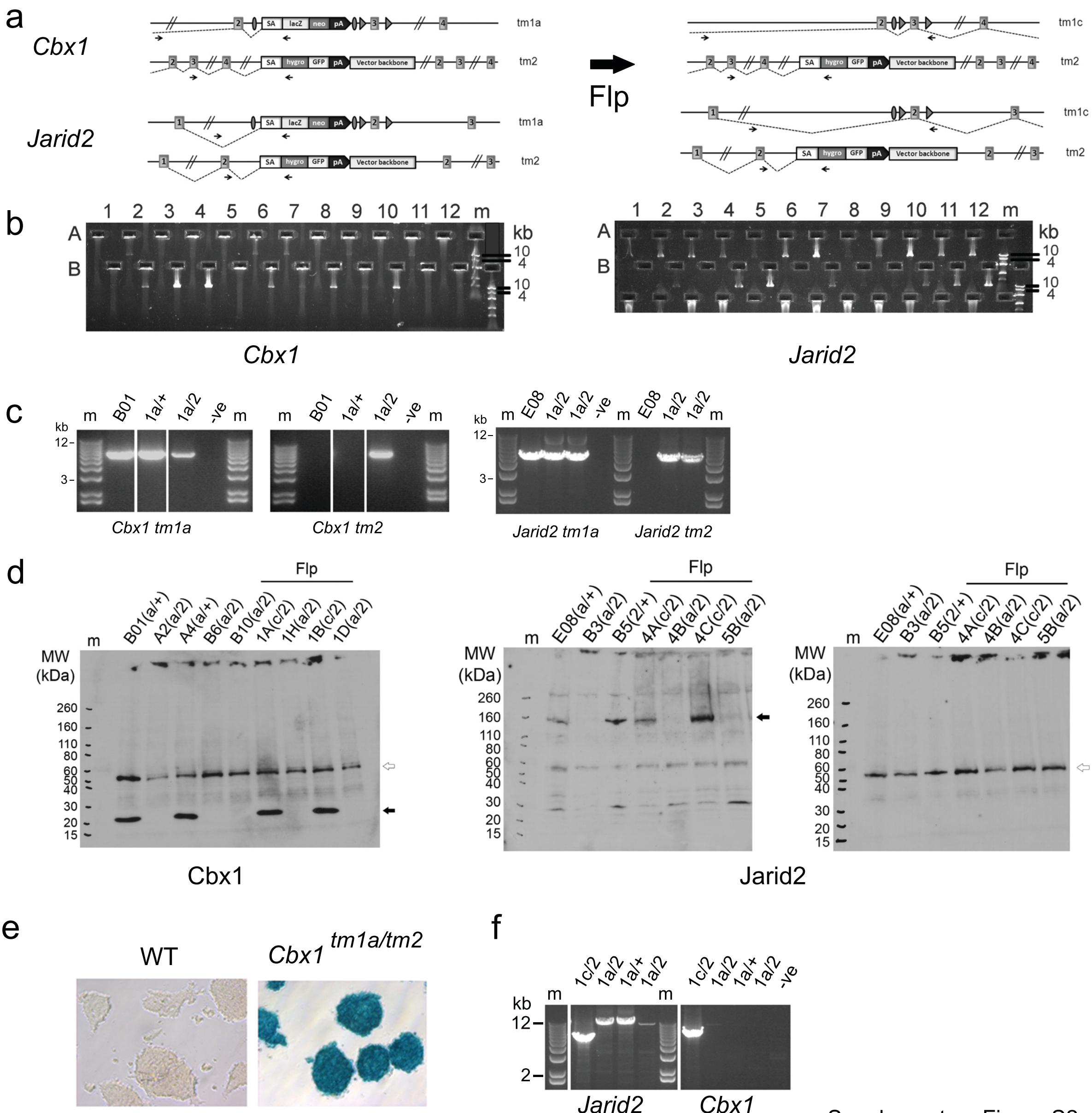

Supplementary Figure S2

Supplementary Figure S2. Examples of gene targeting schematics and validation for *Cbx1* and *Jarid2* bi-allelic targeted and revertant mutant ES cell lines. Shown are details for gene targeting into IKMC heterozygous mutant *tm1a* ('knockout-first') ES cell lines using the pI_hygGFP insertion targeting vector to generate the *tm2* allele, and details on reversion of the *tm1a* allele to the conditional form *tm1c* using Flp recombinase.

(a) Schematic showing pI_hygGFP insertion vector targeted *Cbx1*, and *Jarid2 tm1a/tm2* alleles; and reversion of the *tm1a* alleles to the *tm1c* form after treatment with Flp recombinase. Locations of primers for long-range PCR genotyping on genomic DNA are noted as small arrows below each allele.

(b) Examples of screening long-range PCRs run on E-Gels (Invitrogen) using genomic DNA from ES cell clones generated in second allele targeting experiments with pI_hygGFP vectors electroporated into heterozygous 'knockout-first' *(tm1a/*+) *Cbx1* and *Jarid2* cell lines. Locus specific gap region primers ex5 and universal primer hygro_1R were used to genotype the *tm2* targeted alleles, and generate a product of approximately 6 kb. Each well contains DNA from a single ES cell clone; rows are labelled A, B; columns labelled 1-12. E-Gel images have been cropped to show only the relevant lanes, and the marker lane from the upper row of wells has been blocked over for clarity. Marker lane, m (E-Gel 96 High Range DNA Marker, Invitrogen).

(c) Examples of long-range PCR genotyping run on standard agarose gels for better resolution, using genomic DNA for pI_hygGFP targeted *Cbx1* and *Jarid2* loci. Locus specific external primers gf3 and universal primer LAR_3 (binds to the en2 intron) were used to reconfirm the genotype of the *tm1a* 'knockout-first' alleles, and generate products of approximately 6.5 kb. Locus specific gap region primers ex5 and universal primer hygro_1R were used to genotype the *tm2* targeted alleles, and generate products of approximately 6 kb. PCR schema is shown below each group of products, and the parental cell line/genotype is shown above each lane. Gel images have been cropped for clarity. m, size marker (1 Kb Plus DNA ladder, Invitrogen). ES lines are noted above each lane: B01 and E08, parental heterozygous *tm1a/*+ ES cell lines used for targeting; 1a, *tm1a* allele; 2, *tm2* allele; +, wild type allele; -ve, no template negative control.

(d) Western blots of nuclear extracts from the original *Cbx1* and *Jarid2* heterozygous *tm1a/*+ ES cell lines, examples of heterozygous and *tm1a/tm2* clones obtained after electroporation with the pI_hygGFP vector, and examples of *tm1a/tm2* (non-reverted) and *tm1c/tm2* (reverted) clones obtained after Flp recombinase treatment (as labelled), for *Cbx1* and *Jarid2* loci. Black arrows denote Cbx1 or Jarid2 product from their respective loci; open arrow is alpha-tubulin loading control. Blots have been cropped to show the relevant lanes of the full gel length including the wells that retain some background staining. Marker lane, m (Novex sharp pre-stained protein standard, Invitrogen). ES lines are noted above each lane: *Cbx1*-B01 and *Jarid2*-E08 are the parental heterozygous *tm1a/*+ ES cell lines used for targeting. Other lanes show cell lines obtained post-electroporation with the genotype in brackets: a, *tm1a* allele; c, *tm1c* allele; 2, *tm2* allele; +, wild type allele.

(e) Brightfield microscopic image of bi-allelic targeted ES cells (*Cbx1 tm1a/tm2*) staining positive for X-gal compared to wild type cells which are negative for stain. Images shown at identical magnification factor.

(f) Long-range PCR on genomic DNA is used to genotype alleles that have undergone reversion via Flp recombination. Locus specific external primers gf3 and universal primer LR (binds just 3' to the 3' loxP site of the *tm1a* and *tm1c* alleles) amplify genomic DNA from *Cbx1* (right panel) and *Jarid2* (left panel) *tm1a* or *tm1c* alleles, however the expected product size is reduced from 12 kb (undetectable for the Cbx1 locus) in the *tm1a* allele to approximately 7 kb after Flp recombination (*tm1c* allele). There is no amplification with these primer sets on genomic DNA from the constitutive mutant *tm2* alleles as the LR primer sequence is not present. Gel images have been cropped for clarity. Labels are as in (c).

a

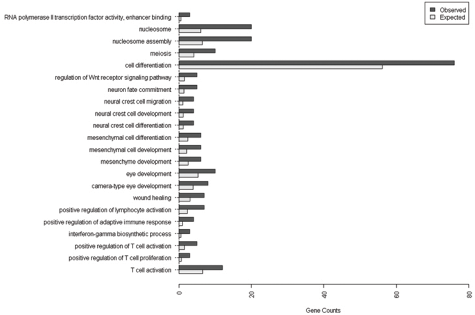

b

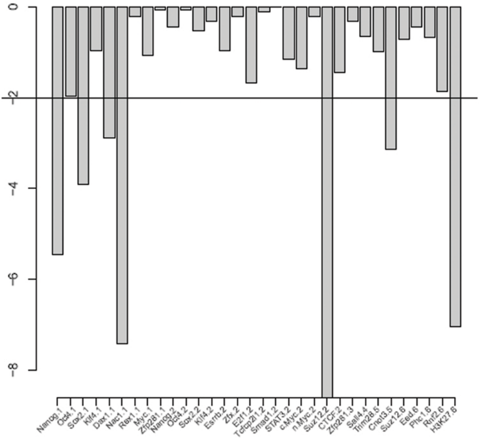

Supplementary Figure S3

Supplementary Figure S3. Analysis of gene expression microarray data of genes with differential expression between *Jarid2 tm1c/2* revertant and *tm1a/2* null mutant ES cells. n=3 biological replicates (independent ES clones) per genotype.

(a) Gene Ontology (GO) analysis of differentially expressed genes upregulated upon *Jarid2* reversion. Categories shown have significantly different gene counts between observed and expected ($p<0.05$).

(b) Statistical analysis of changes in gene expression for target genes of selected transcription factors, chromatin factors, or H3K27me3 marks (using published datatsets as described in the main text) associated with self-renewal and pluripotency regulatory pathways, in *Jarid2 tm1c/2* revertant compared to *tm1a/2* null ES cell lines. Bars represent the p-values (shown on the vertical axis as 10^x) obtained for differential expression (including both up- and down-regulated genes) of the selected target gene groupings versus all genes on the microarray; the horizontal line at 10^-2 indicates an arbitrary statistical significance cutoff of p=0.01 therefore target gene groups with any p-values that cross this line are considered significantly differentially expressed.

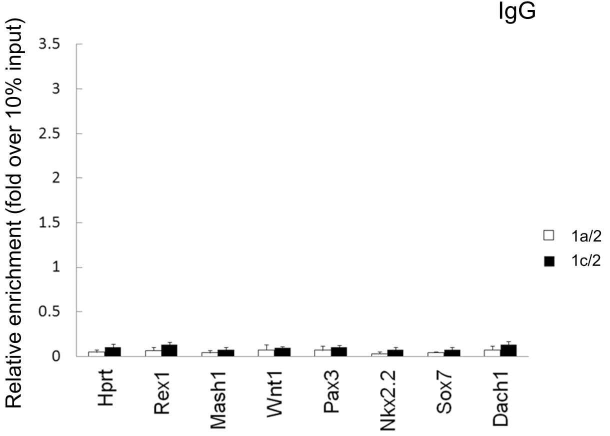

Supplementary Figure S4

Supplementary Figure S4. Functional rescue of Polycomb PRC1 recruitment to PRC2 target genes in *Jarid2* revertant cell lines (1c/2) compared to null lines (1a/2) was shown by restored Mel18 binding at some of the known Jarid2-dependent gene promoter regions, assessed by ChIP-qPCR. IgG is shown here as a control for nonspecific binding. *Hprt* promoter is known to be negative for binding. Results show mean +/- s.d. of three biological replicates (independent cell lines), where values are expressed relative to 10% input chromatin. Differences in IgG binding were not found to be statistically significant, as expected ($p > 0.05$, *t*-test).

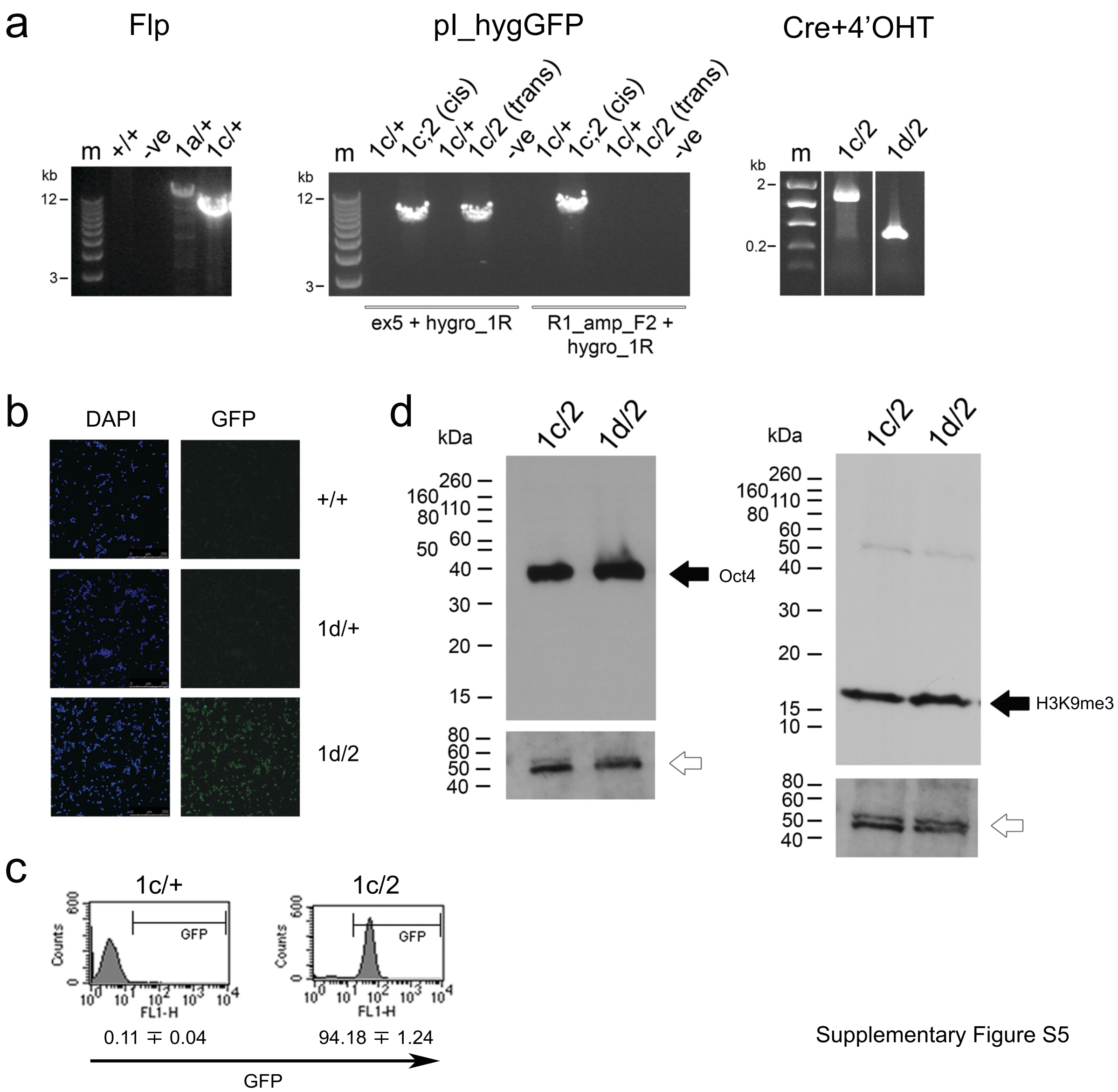

Supplementary Figure S5

Supplementary Figure S5. Genotyping of *Setdb1* inducible conditional mutant ES cells and additional characterization data. Refer to the *Setdb1* allele diagrams and positions of PCR primers as noted in the schematic diagram in **Figure 2a**.

(a) Genotyping of ES cell clones at each step in the process of generating *Setdb1* inducible conditional mutant ES cell lines was done by long range-PCR or short-amplicon PCR on genomic DNA. ES cell clones which have undergone reversion by Flp recombinase are genotyped using the gene-specific primer gf3 with universal primer LR (binds just 3' to the 3' loxP site). PCR on DNA from the original *tm1a* allele generates a product of approximately 12 kb while on the Flp recombined *tm1c* allele template they generate an approximately 6 kb product (Flp panel). ES cell clones following targeting with the pI_hygGFP insertion vector are genotyped using the locus specific primer ex5 and the universal primer hygro_1R however this does not distinguish between *tm1c* allele cis and trans targeting events. To detect undesirable cis targeting events, clones were genotyped using two universal vector-specific primers R1_amp_F2 (binds just 5' to the FRT site within vector derived DNA present on the *tm1c* allele) and hygro_1R: in cis targeted clones this yields an approximately 6 kb band whereas trans targeted templates will not amplify (pI_hygGFP panel). In those cell lines where the pI_hygGFP insertion vector is confirmed to be targeted in trans, the PCR reaction using primers ex5 and hygro_1R can be routinely used to genotype the *tm2* allele. Integration of the *Rosa26-CreERT2* vector in confirmed *tm1c/tm2* bi-allelic targeted ES cell clones was inferred based upon recombination at the *tm1c* allele following Cre induction (thereby generating the *tm1d* allele). PCR on genomic DNA with locus specific primer FOR1 and universal primer LR (which binds to each of the *tm1a*, *tm1c,* or *tm1d* alleles) distinguishes between the original *tm1c* allele template which yields a 1 kb amplicon, and the Cre recombined *tm1d* allele template which yields a 300 bp amplicon (Cre+4'OHT panel).

(b) Fixed *Setdb1* mutant ES cells have detectable GFP expressed from the *tm2* allele. Fixed cells (after 48 hour 4'OHT treatment) were analyzed by fluorescence microscopy; nuclei were stained with DAPI (blue) for orientation, GFP antibody signal is green and is also localized to the nucleus. Genotypes are indicated along the right hand side. Scale bar (shown on DAPI images at bottom right) is 250 μm; GFP images were taken at the same magnification.

(c) Live *Setdb1* mutant ES cells express GFP from the *tm2* allele. Live unstained cells were analyzed by FACS; a representative histogram and the average percentage of cells expressing GFP and standard deviation of three independent cell lines for each genotype is shown.

(d) Western blots of *Setdb1* mutant ES cells (*tm1d/tm2*) performed 4 days after the start of a 48 hour 4'OHT treatment show no change in global levels of histone H3K9me3 or Oct4 (black arrows), compared to *Setdb1 tm1c/tm2* controls. Loading control is alpha-tubulin (open arrows).

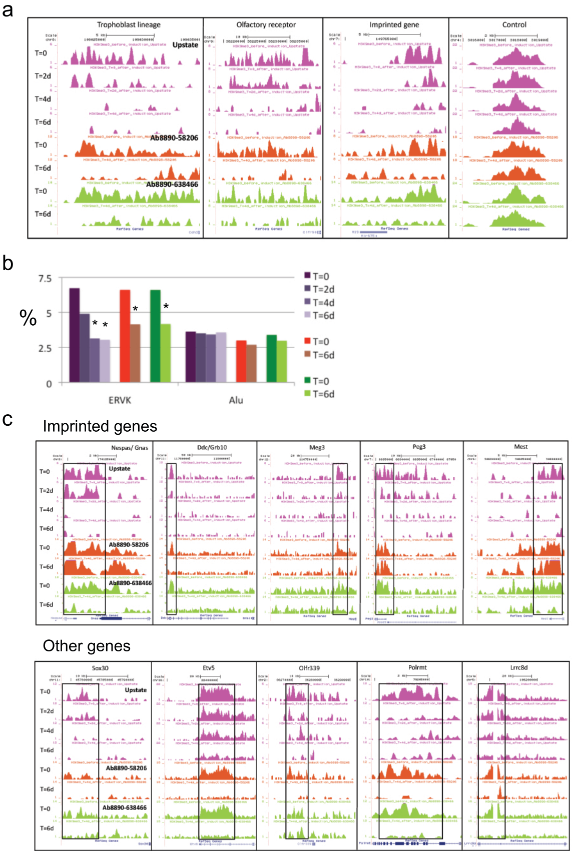

Supplementary Figure S6

Supplementary Figure S6. Analysis of H3K9me3 ChIP-seq data obtained with all three antibodies used in this study (Upstate 07-442-4861, purple; Abcam Ab8898-58206, orange; Abcam Ab8898-638466, green) of *Setdb1 tm1c/tm2* mutant ES cells at timepoints before and following a 48 hour 4'OHT treatment (and conversion to *Setdb1 tm1d/tm2)*. T=0, untreated cells at start of timecourse; T=2d, 2 days after start of 4'OHT treatment; T=4d, 4 days after start of 4'OHT treatment; T=6d, 6 days after start of 4'OHT treatment.

(a) H3K9me3 ChIP-seq profiles for the same genomic regions (trophoblast marker *Cdh3*, olfactory receptor *Olfr901*, and imprinted gene *H19*) as shown in Figure 3, before and after *Setdb1* ablation.

(b) The percentage of ChIP-seq reads present on endogenous retrovirus repeats (ERVs) or Alu repeats during ablation of *Setdb1*. * indicates sample significantly different to T=0 sample, $p<0.01$ (Fisher's Exact test) with the additional requirement of >1.5 fold change between the profiles.

(c) H3K9me3 ChIP-seq profiles for selected further examples of previously defined Setdb1 targets including imprinted genes (top panel), showing reduction of H3K9me3 after *Setdb1* ablation.

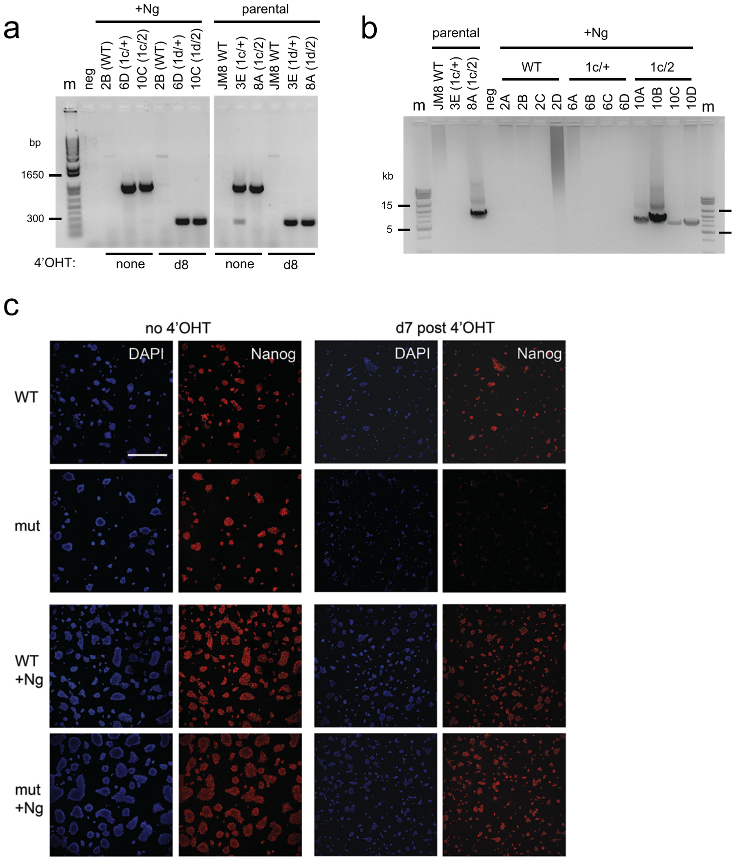

Supplementary Figure S7

Supplementary Figure S7. Genotyping of the two *Setdb1* mutant alleles in wildtype (WT) control and *Setdb1* mutant ES cells with a Nanog transgene (+Ng) added, and Nanog protein expression in ES cells. Refer to Supplementary Figure S5 for further details on genotyping strategies used.

(a) Short amplicon PCR using primers Setdb1_FOR1 and LR to confirm the *Setdb1 tm1* mutant allele genotype before (none) and 8 days (d8) after the start of 4'OHT treatment, for parental ES cell lines and those derived from them that are +Ng.

(b) Long-range PCR using primers Setdb1_ex5 and hygro_1R confirms the presence of the *Setdb1 tm2* allele in each of four independent Setdb1 tm1c/tm2 +Ng lines.

(c) Immunofluoresence staining with Nanog antibody in JM8 wildtype (WT) ES cells and *Setdb1 tm1c/tm2* (mut) ES cells (which become *tm1d/tm2* following 4'OHT treatment), before 4'OHT treatment and 7 days following 4'OHT treatment, comparing parental cell lines to those derived from them that are +Ng. Representative example from 4 independent experiments shown. Scale bar, 500 μm.

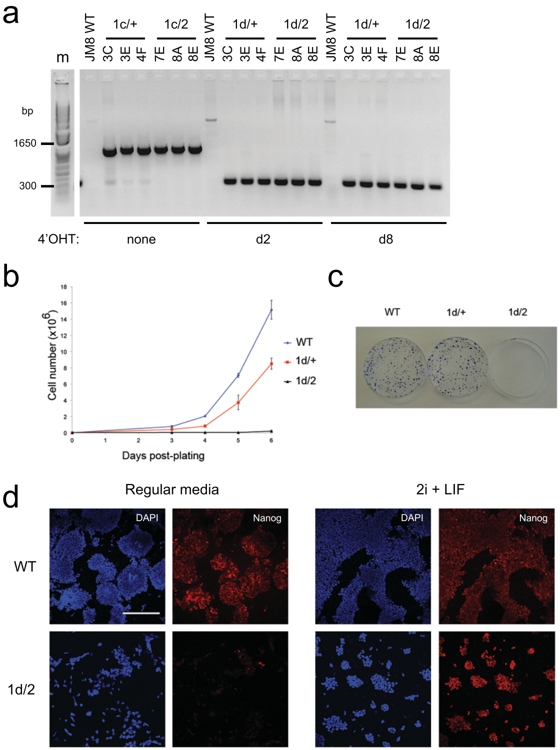

Supplementary Figure S8

Supplementary Figure S8. Enhancing naïve pluripotency-promoting conditions by culturing inducible conditional *Setdb1* mutant ES cell lines in 2i+LIF media.

(a) Short amplicon PCR using primers Setdb1_FOR1 and LR to confirm the *Setdb1 tm1* mutant allele genotype before and after 4'OHT treatment (d2, day2; d8, day8 post-treatment), for ES cell lines grown in 2i+LIF media. Cell line identifiers are written below the horizontal black line at the top of the gel images, while the corresponding *Setdb1* allele genotype is written above. m, 1 Kb Plus DNA Ladder (Invitrogen). neg, no DNA negative control reaction.

(b) Growth of *Setdb1 tm1d/tm2* (1d/2) mutant ES cells grown in 2i+LIF remains compromised, while control wildtype (WT) JM8 ES cells show robust growth characteristics, and *Setdb1 +/tm1d* (1d/+) control cells show an intermediate growth phenotype. Cells were plated following a 4 day 4'OHT treatment and counted at the time intervals indicated. Points represent the mean and error bars indicate the s.d. of 3 biological replicates.

(c) *Setdb1 tm1d/tm2* mutant ES cells grown in 2i+LIF are unable to form normal colonies of undifferentiated ES cells, whereas control wildtype JM8 and *Setdb1 +/tm1d* ES cells form colonies. Cells were plated at low density onto 10cm plates following a 4 day 4'OHT treatment and stained with methylene blue after 10 days. Representative examples are shown; experiment was repeated 3 times.

(d) Immunofluoresence staining with Nanog antibody in JM8 wildtype (WT) ES cells and *Setdb1 tm1d/tm2* (1d/2*)* ES cells grown in regular ES cell media or 2i+LIF media for 8 days after 4'OHT treatment. Representative example from 3 independent experiments shown. Scale bar, 250 μm.

Supplementary Table S1. Details on Intermediate Vector designs from the IKMC (EUCOMM and KOMP-CSD) targeting pipeline resources used in this study and insertion targeted trapping vector construction. The reading frame of the splice acceptor site of the 3' exon, used to generate the insertion targeted trapping vectors (pI_hygGFP) in the 3-way Gateway reaction are shown.

Targeted gene	Targeting pipeline resource	Vector design ID	Intermediate Vector ID	Reading frame for pI_hygGFP vector
Brd8	EUCOMM	39779	PCS00019_A_H09	2
Cbx1	EUCOMM	39792	PCS00019_A_B11	2
Ddx27	KOMP-CSD	35616	PCS00023_E_A02	2
Dot1l	KOMP-CSD	47359	PCS00013_A_G05	0
Epc2	EUCOMM	47364	PCS00013_A_A01	1
Ing3	EUCOMM	39832	PCS00019_A_E05	0
Jarid2	KOMP-CSD	47358	PCS00013_A_H05	1
Kdm4c	KOMP-CSD	36279	PCS00023_E_C01	0
Mier1	EUCOMM	39842	PCS00019_A_B08	0
Phf20	EUCOMM	39870	PCS00019_A_D11	1
Setdb1	EUCOMM	39882	PCS00019_A_G05	1
Smyd5	EUCOMM	39894	PCS00019_A_E02	1
Supv3l1	EUCOMM	39898	PCS00019_A_G06	1
Kmt5b	KOMP-CSD	47356	PCS00013_A_E03	2

Supplementary Table S3. PCR primers used for genotyping of mutant ES cells. Locus specific forward primers gf3 and universal reverse primer LAR_3 were used to reconfirm the genotype of the *tm1a* 'knockout – first' allele except for the *Ing3* locus where universal forward primer RAF_5 was used in combination with locus specific reverse primer gr3. Locus specific primers ex5 (located within the deletion region) and universal primer hygro_1R were used to genotype the *tm2* second targeted allele. Universal reverse primer LR and locus specific primers gf3 were used to genotype Flp mediated recombination events. Universal primers R1_amp_F2 and hygro_1R were used to genotype cis targeting of the pL3L4_hygGFP vector into *tm1c*/+ ES cells, and universal primer LR with locus specific primers FOR1 were used to genotype Cre mediated recombination events converting the *tm1c* to *tm1d* alleles. Primers are written in the 5' to 3' direction.

Targeted gene	gf3 primer	ex5 primer
Brd8	GCAGCCTACAGCTTACCGGTTCTG	GCCGGGATTACTACTCCACAGTAG
Cbx1	GAATGACCACACTTCCTCTTACGG	GAGACAGATAAGTCAGAGGGAGGC
Ddx27	GAGTCGGACTCCGGAGACGAGGAG	TGCCCTGTCTGACTGTAACTTTTC
Dot1l	TCCAGAACATTGGTGTCCAACCAC	AGGATCTGCCCACACCCATCAG
Epc2	ATTCAGAATGAGGTCATTGGGTTTG	CAGCCCAAGCAGTTCATTCATATTC
Ing3	Not used; see below	GGCAGTCACATTGGGACCCTCTAG
Jarid2	AGGTATTTTGTTCGCCGTGTGTTTC	TCTTTCTGTGCCTTGTTCTGACCTG
Kdm4c	GAAGTAGGAGGAGAACCATAGTTCCAGAG	AAGAGGTAAGAAGCCCATCACATC
Mier1	GAAGACTGACCTCCAGAAAATCTG	CAGGTCCGTGAGTAAGGATAACGC
Phf20	GTGACCCTGCTCAGGCTGGCAGGC	CGTTGGAACCATCGTTACGATGAG
Setdb1	CGCCTGTGCGCGTTATTCCGAGTG	GTTCTCTAGATACAGCATGTCTTG
Smyd5	GCGGCCTCCATGTGCGACGTGTTC	GTGAGCACATCTTGCCTGCTTCAG
Supv3l1	CTAGATGGTGTTGGGGACCTCTCG	GAGTGAGCGTGCTAATGTATGATG
Kmt5b	CCAGATGAGCTCAGATCAGTGTCTTG	TAGCCCTGTCTGTACTGGCTCTGTG
**Other primers**		
***Ing3*_gr3**	GCACACATCAGTGGACCTTCTGAG	
**RAF_5**	CACACCTCCCCCTGAACCTGAAAC	
**LAR_3**	CAACGGGTTCTTCTGTTAGTCC	
**hygro_1R**	GCAGCTATTTACCCGCAGGA	
**LR**	ACTGATGGCGAGCTCAGACC	
***Setdb1*_FOR1**	GAGTGGAGTTGACCAGCACGAG	
**R1_amp_F2**	AAGGCGCATAACGATACCACGATATC	

Supplementary Table S4. Oligonucleotide primers for recombineering to generate the Intermediate Vectors from the EUCOMM and KOMP-CSD gene targeting resources used in this study. The locus specific recombineering primers which define the 5' and 3' ends of the genomic homology arms of the Intermediate Vectors are noted as G5 and G3 primer respectively. The U3 and D5 primers mark the 5' and 3' boundaries respectively of the loxP flanked region in the 3' genomic homology arm, which is deleted by Cre recombinase to make the final pI_hygGFP insertion targeted trapping vectors, and is critical to the genotyping strategy used. Primers are written in the 5' to 3' direction, and are organized left to right in corresponding order within the genomic locus.

Targeted gene	G5 primer	U3 primer	D5 primer	G3 primer
Brd8	GACCAAAGCAAAATGAGATTCTCCACGGTTAATGACATACATGTATATAT	TATTGAATACACATTTTTAGAACATCTTGTTTAAATGCCACGGCTTTAAG	TACTTAGTCCTGGCTAGCTTGATTGCACATTCCAGTACTAGGAAGGCAGA	TCTGCTTTTCAAATAAATAATACCAGGCATAGTGGAGGTCTGTAATTCTA
Cbx1	TGGTTTTCAGATAATTACCCCTTAGTTAAAGACAATGTATAATGAATTAG	AGGACCTACATGCAGAGTATTATATGACTGAGTTTTTTGTCTTGTTTTAA	TGGCCTATAATTTTGTATCCCAAGTTTGCCTTAGACTTGTAGCAATTCTC	TCCTCTTTCCTAGTTTGTTTCTGGAATTTAGACTTTAAAAGCCCCATTCC
Ddx27	GCTTGTTGGTATCCAATTCTGTATCAGTTTTGTGTTTCCTTTTGAAACTG	TTTTTTGCACTTGCTGTTTCCTCCGATTGAAAGACTTCTTCTCTAGAATT	ACGTGGATCTGAGTTTGAAGCCAGGTTTGTCTACGCTGAGCATTTCAGGG	AATAAATAAGAAAGGTAGCCATCTCCTGCTGGATACTCTAGAGAGTAATG
Dot1l	TTGGACACCAATGTTCTGGAAAGATAACCAGCTTGGCTTGCATAACGAGG	GACTAGAGGGGAAAAGAACAAGGAAACAGAAGACGCAGCACTCCGGCTTC	AGAGGCATCTCTCCATGACCCTTGTCTGGGAAAACCTCAGTTGTTTGTTT	TGGGTGGTTGTGTGAGTCCTTGAGTTAAGATATTTGTACAGCTCTGTGCA
Epc2	TAATAGCCTGTTTTATCTTTAAATTTTACTTATTATATGTTTATGGTATATGTATGTGAGTACATATTAC	TTCATCACCACTACCACATTAAGAAGAAAATATTCAAGTCACAGGAAATAAGTCTGTGTTATAAATTTTA	GAATGTTAAACATGAGATATTTAAATCTGTTTGTATTTTCATTTGAGCTTGGAGATCACTTTGAGTTTTA	TTTTAAGACCATTAAAACATATAAAACAAAAAATTGAGGTTGGCTGTAGAAAACATGTCACCTATTGATA
Ing3	ACTACTCTTAATGCAGTATCTGCTAAAAACAATTTTCTAAAAATCTGGGT	CTATGGAGCAAGTGTTGACTCTGTCATCACTATTTTTATTAAAAGAAGTA	GGGTCACCAAAAGCCATCGGAAAACACATTACGATTCAGAAATAATCTCA	GAACATTTCTTCAATTATTGCTATGCCATTATCTCTTCCTGTCAGGTATA
Jarid2	ACTACTATTCAAGGATGAGAAGAAATAGTGTATGCAACTTTAACCCTCAT	AATTTTGCTACTATTATGAATCATAGTATAAATATAATGTTTTTCCTATG	TATTGTATTTATAGATATATTTTTATGTTTCTTTACAAAATAATATTTGG	AATGTACATTTGGTAGAGGTTTTGTTGAGTCACATAGGCATTTATTCTAT
Kdm4c	ATGTGATTGTATGTTTGGGTTTGTGTGACCAGCATCTTTATCTTCTGAGA	TCTATATAGTATTGGTACTTTGGAATTTTGTTGCATATCTGAGATAGTAA	ACATAGTATATTACTGAAATTTTGTGTGTTTAACTTTTGAGTCGGTGTTT	GGGGCTTAAAAATATCTCACAATTTAATGGTTTCCAAATAACAGAATGTT
Mier1	GAACACTGCATGTCTATCACCAGATCTTTTTTATGTTTAAAATAGGAAAT	GAAAAAAAGTTATGGCTGCCACTTCTGTGAGTAAAGGCACTCTCATCAAA	CAGAAACCTTTCTTTGCCTGCATATACTTTTTGTAATGATTTTTAAAAGT	TCTGGATTTCCTTACTGCCTTTTGATACACATACTGTACTCTGTTCTTCA
Phf20	CCCTTCTCTGCCTTTTGCTTTTCTGAATCCTACTCTAATTACAGAGTCTT	ATGCCTGGTTTTAAAGTGTAGGACTTTGGCACTAGAGAGATGCTTACATC	CTCCTCTAGAGTTCAGTTTTCACCTGTTTGGTGCAATTGATGTAATAGTT	ACTTTGTTTTCAGTTCTGGGTGTTAAATAAATTCAAGGCTTCATTTGTGT
Setdb1	CAATTTTCCACCTGCTGTTACAAAAGTCCTAATATCTGATATTTATTATC	GAGACCAGTTATACTCACACAAACACTCCCCCACAGACAGATTTTGTTTT	ATACCTTGGAGATACAGGCAGCTGAAAGACTCTCAACATGGGTGCTGGAA	GATAGATTTGAGTTTAATTCTTAGACTATACATACGAAAGTTAGCGATGG
Smyd5	TTTGCATTCCCCTGGGTAAAAGTCAGGTTAAATAATCATAATAACAACAT	GTGTGCTCGCGCCATCTCTGTATGTAGATGTACATGCCTGTGTATGTGAA	CTGCGATGGGAGGTGCCTCCAAAATGCTCCTTGGGAAGGATTCTCTCTTA	ATGGTTAGACCTGTGTTTCTTTGTTTCTTTGGAAATTTTCTTGGAAATCA

Supv3l1	ACCACAAAGTATGCTCTGTGTACCTGGTTAAAGTTTGATCTTACTCCTAT	AGGAGATAAACACACACAGCCTGGACATTAGAGGGACAGGAACTTCTTTT	TCCAACAGGAGGAAAGGAACACACCAGTAACATGTCTGAGAGAACCAATT	TAATATATTCACCTGAGTGCATACCCTACATATAAATAACAAAATCTGAA
Kmt5b	TATTATCTAGAAGTGTCTACCCATGTTACAAATGACTTTCTCAATCTCTGCTTTTCCTAACGTTAGTCAG	GATGCAGACAGTCATCAAGAAATGTCATAATGTGGATCCCAAGAACCAAAGGGTCTCAAGGAATTAAGTT	TCTTGCACTGGGGGTGGGGAGTCAGATCTTTCCCATGGCTTCATTATGGGGTCTTACTGTTTTAAATAAT	TAATATTAATATGTATCTGAAGGGTCACTGAAGTACTGTCTCCTTTGCCTTTTGTATTTTTCTTACCTTT
